# Green-Synthesized Mesoporous Silica-Enhanced Herbal Nanofibers with Antimicrobial, Anti-Inflammatory, and Biocompatible Wound Healing Potential

**DOI:** 10.3390/molecules31183257

**Published:** 2026-09-14

**Authors:** Hebah Ayash, Gülşah Esen, Ece Sabuncu, İnci Deniz, Pınar Akkuş Süt, Gökçen Yaşayan, Hande Sipahi, Ahmet Aydın, Soodabeh Davaran, Muhammed Hamitoğlu

**Affiliations:** 1 Pharmacological and Diagnostic Research Center, Faculty of Pharmacy, Al-Ahliyya Amman University, Amman 19328, Jordan; 2 Department of Pharmaceutical Toxicology, Faculty of Pharmacy, Yeditepe University, 34755 Istanbul, Türkiye; gulsah.esen@yeditepe.edu.tr (G.E.); ece.sabuncu@std.yeditepe.edu.tr (E.S.); hande.sipahi@sbu.edu.tr (H.S.); ahmet.aydin@yeditepe.edu.tr (A.A.); 3 Yağmur Sönmez Microbiology R&D Laboratory, Department of Pharmaceutical Microbiology, Faculty of Pharmacy, Yeditepe University, 34755 Istanbul, Türkiye; inci.deniz@yeditepe.edu.tr; 4 TEM & SEM & AFM Imaging Laboratory, Department of Genetics and Bioengineering, Yeditepe University, 34755 Istanbul, Türkiye; pinar.akkus@yeditepe.edu.tr; 5 Department of Pharmaceutical Technology, Faculty of Pharmacy, Yeditepe University, 34755 Istanbul, Türkiye; gokcen.yasayan@yeditepe.edu.tr; 6 Department of Pharmaceutical Toxicology, Hamidiye Faculty of Pharmacy, University of Health Sciences, 34668 Istanbul, Türkiye; 7 Drug Applied Research Center, Tabriz University of Medical Sciences, Tabriz 51656-65811, Iran; s.davaran2018@gmail.com; 8 Engineered Biomaterial Research Center, Khazar University, AZ1096 Baku, Azerbaijan

**Keywords:** electrospinning, *Hypericum perforatum*, mesoporous silica nanoparticles, green synthesis, anti-inflammatory, wound dressing, genetic safety

## Abstract

Advanced wound dressings should actively promote tissue repair while preventing microbial infection and excessive inflammation. In this study, multifunctional electrospun nanofibers composed of poly(vinyl alcohol)/chitosan/gelatin were developed by incorporating green-synthesized mesoporous silica nanoparticles (MSNs), Acacia gum, and *Hypericum perforatum* extract (HPE). Biological activity and safety were evaluated using disk-diffusion antimicrobial testing, MTT cell viability assays, nitric oxide measurement in LPS-stimulated RAW 264.7 macrophages, an L929 fibroblast scratch assay, and Ames and comet assays. The optimized nanofibers exhibited a uniform morphology with an average fiber diameter of approximately 400 nm and high encapsulation efficiencies (86–96%). MSN incorporation significantly prolonged hypericin release, producing a biphasic release profile with approximately 1.4-fold slower release than nanofibers without MSNs, while increasing Young’s modulus from 2.1 to 2.5 MPa. The hybrid nanofibers demonstrated broad-spectrum antimicrobial activity against Gram-positive bacteria, Gram-negative bacteria, and *Candida albicans*, achieving approximately 70% of the efficacy of standard antimicrobial agents. In lipopolysaccharide-stimulated RAW 264.7 macrophages, the optimized formulation reduced nitric oxide production by approximately 51%, comparable to indomethacin and L-NAME. Although fibroblast migration was not significantly enhanced, the nanofibers exhibited acceptable cytocompatibility and showed no mutagenic or genotoxic effects in Ames and comet assays. These findings demonstrate that green-synthesized MSN-enhanced herbal nanofibers represent a safe and promising platform for sustained drug delivery and advanced wound-healing applications.

## 1. Introduction

Wound dressings are crucial for protecting injured tissue, managing exudate, and creating a pro-healing microenvironment. Burns and chronic ulcers remain major burdens worldwide [1]. While conventional gauze is inexpensive, it lacks advanced functionality; modern dressings (films, foams, hydrogels, nanofibers) offer superior performance. An ideal dressing is biocompatible, absorbs exudate, prevents microbial invasion, and supports regeneration, while being easy to apply and remove [2].

Electrospun nanofibers are promising because they mimic extracellular matrix architecture and provide high surface area, tunable porosity, and capacity for loading therapeutics [3,4,5]. Consequently, electrospun nanofibers have attracted considerable attention as multifunctional wound dressings and localized drug-delivery systems.

Natural polymers such as chitosan and gelatin are widely used in electrospun scaffolds because of their excellent biocompatibility, biodegradability, and intrinsic biological activity [6]. Chitosan (CS) possesses antimicrobial and hemostatic properties, whereas gelatin (G) promotes cell attachment and proliferation. Poly(vinyl alcohol) (PVA) is commonly incorporated to improve electrospinnability and mechanical stability [7]. Furthermore, natural additives such as acacia gum (AG) can further enhance the structural integrity and biological performance of polymeric nanofibers through improved viscosity, water retention, and bioadhesion [8,9].

Incorporating herbal extracts and MSNs into nanofibers can add antimicrobial and anti-inflammatory functions with controlled release, and can improve mechanical/thermal properties, supporting roles as both dressings and drug-delivery systems [6,10,11,12].

Among inorganic nanomaterials, mesoporous silica nanoparticles (MSNs) are attractive due to their well-defined mesopores (2–50 nm), high surface area, and adjustable surface chemistry [13]. Beyond serving as efficient drug reservoirs, MSNs have been reported to promote hemostasis, enhance cell adhesion, proliferation, and differentiation, and act as carriers for diverse agents, enabling sustained and targeted release while reducing side effects. Functionalization with antimicrobial agents can further prevent infection and modulate inflammation, key to effective healing [14,15,16,17]. Synergistic combinations of plant extracts with MSNs are increasingly explored for accelerated wound repair [18,19].

*Hypericum perforatum* L. (St. John’s wort) has long been employed in traditional medicine, especially in Türkiye, for the treatment of burns and skin injuries because of its antimicrobial, antioxidant, anti-inflammatory, and tissue-regenerative properties. These biological activities are primarily attributed to its major constituents, including hypericin, hyperforin, flavonoids, and phenolic compounds. Nevertheless, the clinical application of these phytochemicals remains limited by poor stability and uncontrolled release, making encapsulation strategies particularly attractive [20,21,22].

Recent research has increasingly focused on combining herbal bioactive compounds with nanostructured carriers to achieve synergistic therapeutic effects. However, the integration of green-synthesized mesoporous silica nanoparticles [23] with *Hypericum perforatum*-loaded electrospun nanofibers has not previously been reported. In addition, studies that simultaneously evaluate antimicrobial efficacy, anti-inflammatory activity, controlled drug release, cytocompatibility, mutagenicity, and genotoxicity in a single wound-dressing platform remain scarce.

Accordingly, this study aimed to develop a sustainable multifunctional nanofibrous wound dressing by incorporating plant-derived mesoporous silica nanoparticles and *Hypericum perforatum* extract (HPE) into electrospun PVA/CS/G nanofibers containing AG. The resulting scaffolds were comprehensively characterized with respect to morphology, physicochemical properties, mechanical behavior, encapsulation efficiency, and drug-release kinetics, followed by biological evaluation including antimicrobial activity, anti-inflammatory efficacy, cytocompatibility, cell migration, mutagenicity, and genotoxicity. This integrated approach provides a comprehensive assessment of both therapeutic performance and biosafety, supporting the potential application of these green nanofibers as advanced wound-healing biomaterials.

## 2. Results

### 2.1. Characterization of Green-Synthesized Mesoporous Silica Nanoparticles

The physicochemical characteristics of the green-synthesized mesoporous silica nanoparticles (MSNs) were evaluated before their incorporation into the electrospun nanofibers. Dynamic light-scattering analysis revealed two particle-size populations centered at approximately 150–190 nm and 500–530 nm, indicating a heterogeneous but nanoscale size distribution. The nanoparticles exhibited a negative surface charge with a zeta potential of −24.1 mV, suggesting good colloidal stability [13] (Figure S1a,b). DLS analysis showed a predominant particle-size population in the range of 150–190 nm, corresponding to the main hydrodynamic size distribution of the MSNs. A secondary population at approximately 500–530 nm was also observed, which is attributed to a limited degree of nanoparticle agglomeration/aggregation in the aqueous dispersion. Since DLS measurements are highly sensitive to larger scattering species, even a relatively small fraction of aggregates can generate a detectable secondary population (Figure S1a).

The FTIR spectrum confirmed the characteristic silica framework by the presence of absorption bands at approximately 470 cm^−1^ (Si–O rocking), 1100 cm^−1^ (asymmetric Si–O–Si stretching), and 1640 cm^−1^ (adsorbed water bending), and a broad band around 3500 cm^−1^ corresponding to surface hydroxyl groups [15] (Figure S1c).

**Figure 1:**
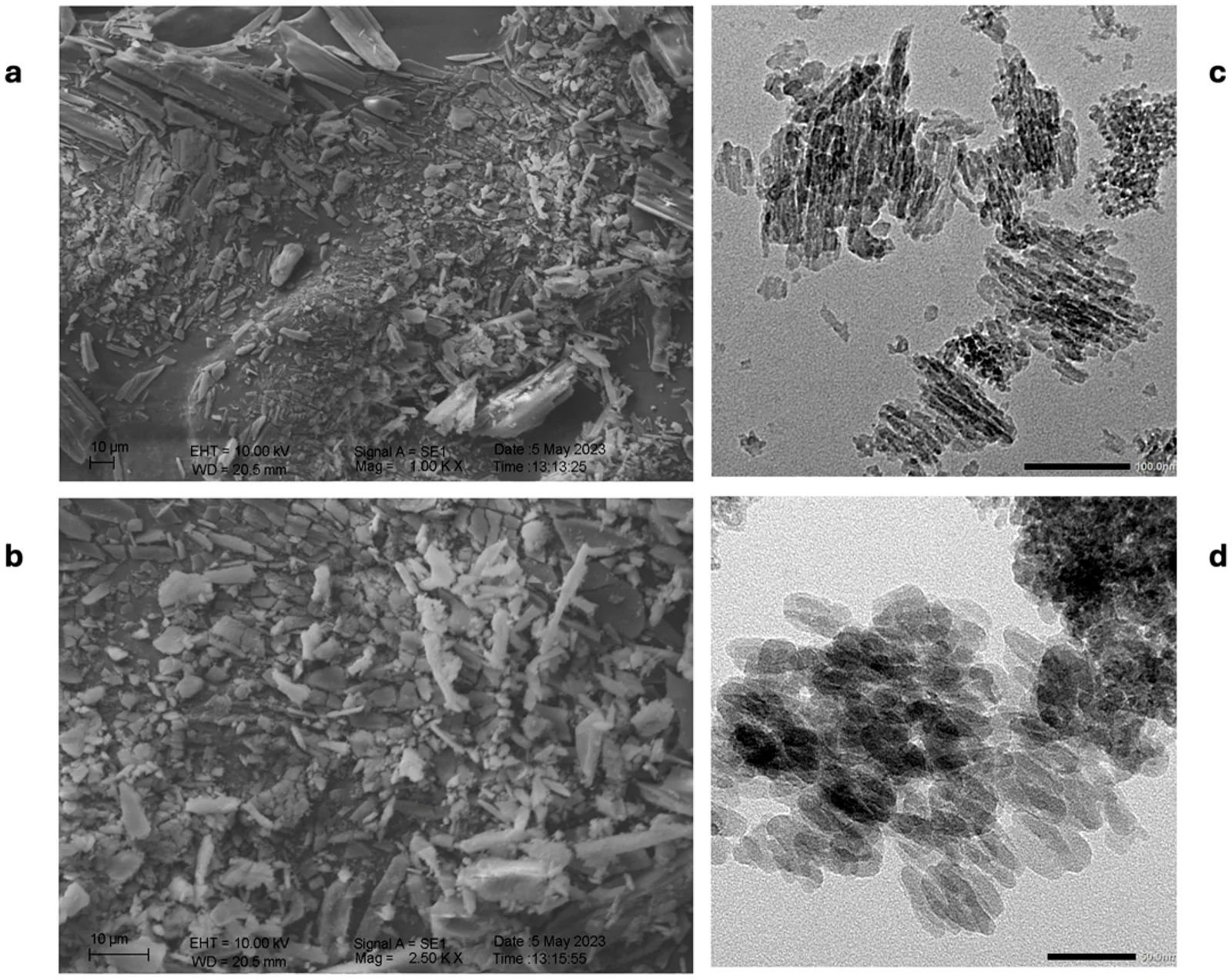
SEM images of MSNs (**a**) at 1K× and (**b**) at 2.5K×. TEM images of MSNs (**c**) at 60K× and (**d**) at 100K×.

The morphology of the synthesized MSNs was further examined by SEM and TEM. SEM images demonstrated predominantly cylindrical particles with relatively uniform morphology (Figure 1a,b), while TEM observations confirmed the mesoporous architecture characteristic of silica nanoparticles (Figure 1c,d). These findings verified the successful synthesis of plant-derived MSNs suitable for drug-loading applications.

### 2.2. Optimization and Characterization of Electrospun Nanofibers

#### 2.2.1. Optimization of Polymer Composition

The different PVA/CS/G formulations were prepared to identify the composition capable of producing continuous electrospun nanofibers with minimal bead formation. Variations in polymer concentration and blend ratio markedly affected solution spinnability and fiber morphology (Figure S2). Increasing the PVA content improved solution viscosity and promoted the formation of continuous fibers, whereas excessive chitosan resulted in unstable jet formation and pronounced bead defects. Incorporation of gelatin further enhanced fiber uniformity by improving chain entanglement within the polymer solution. Among the investigated formulations, the blend containing 12% PVA, 3% CS, and 10% G at a polymer ratio of 70:15:15 (M16) produced the most uniform nanofibers. The incorporation of 1% (*w*/*w*) of AG into formulation M16 improved fiber morphology and structural stability. Therefore, this formulation was selected for subsequent drug-loading experiments. Optimal electrospinning was achieved at an applied voltage of 21.5 kV, a flow rate of 0.7 mL h^−1^, and a tip-to-collector distance of 15 cm. Under these conditions, a stable Taylor cone was maintained throughout the electrospinning process, resulting in smooth, continuous, bead-free nanofibers with a uniform diameter distribution (Table S1).

#### 2.2.2. Morphological and Mechanical Characterization of Optimized Nanofibers

**Figure 2:**
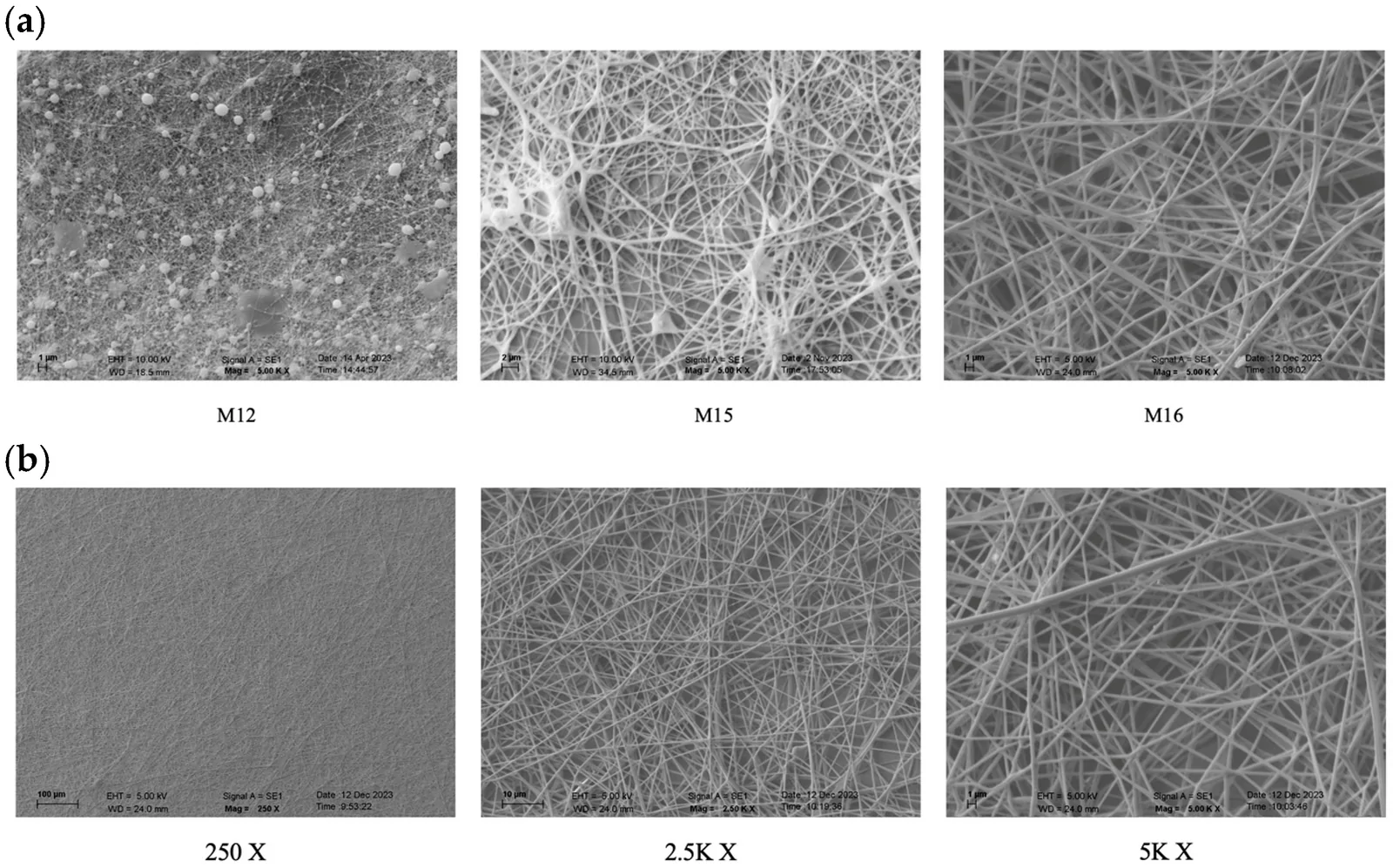
(**a**) Representative SEM images at 5K× of formulations M12, M15, and M16. The addition of 1% (*w*/*w*) acacia gum significantly enhanced morphology in M16. (**b**) SEM images of final electrospun nanofiber formulation (M16) at different magnifications.

The morphology of the optimized electrospun nanofibers was examined by scanning electron microscopy (SEM). As shown in Figure 2 and Figure S2, the optimized formulation (M16) consisted of smooth, continuous, randomly oriented nanofibers with negligible bead formation. The optimized electrospinning conditions produced a homogeneous fibrous network with uniform diameter distribution, indicating stable jet formation throughout the electrospinning process.

**Figure 3:**
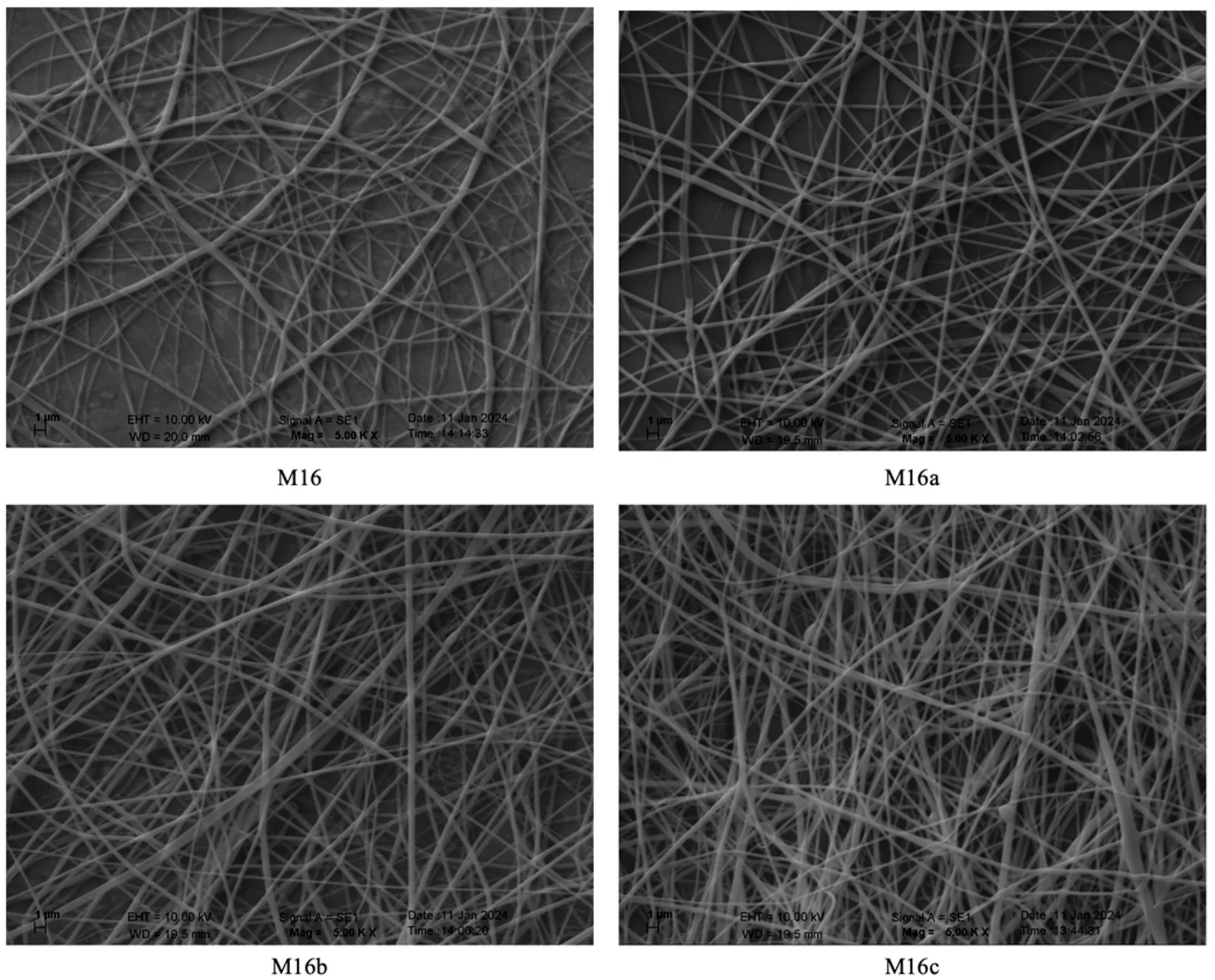
SEM images of M16, M16a, M16b, and M16c nanofibers at 5K×.

The incorporation of MSNs (M16a), HPE (M16b), and the combined MSNs–HPE formulation (M16c) did not adversely affect fiber continuity or overall morphology. All loaded formulations retained a smooth surface and bead-free structure, demonstrating that the incorporation of bioactive components did not compromise the structural integrity of the electrospun scaffold (Figure 3).

Fiber diameter distributions were determined from the SEM images using ImageJ, and the corresponding results are presented in Figure S3. Approximately 100 individual fibers were measured for each sample. The mean fiber diameters ± SD were 382 ± 52 nm for M16, 415 ± 59 nm for M16a, 419 ± 54 nm for M16b, and 417 ± 64 nm for M16c (Figure S3).

**Figure 4:**
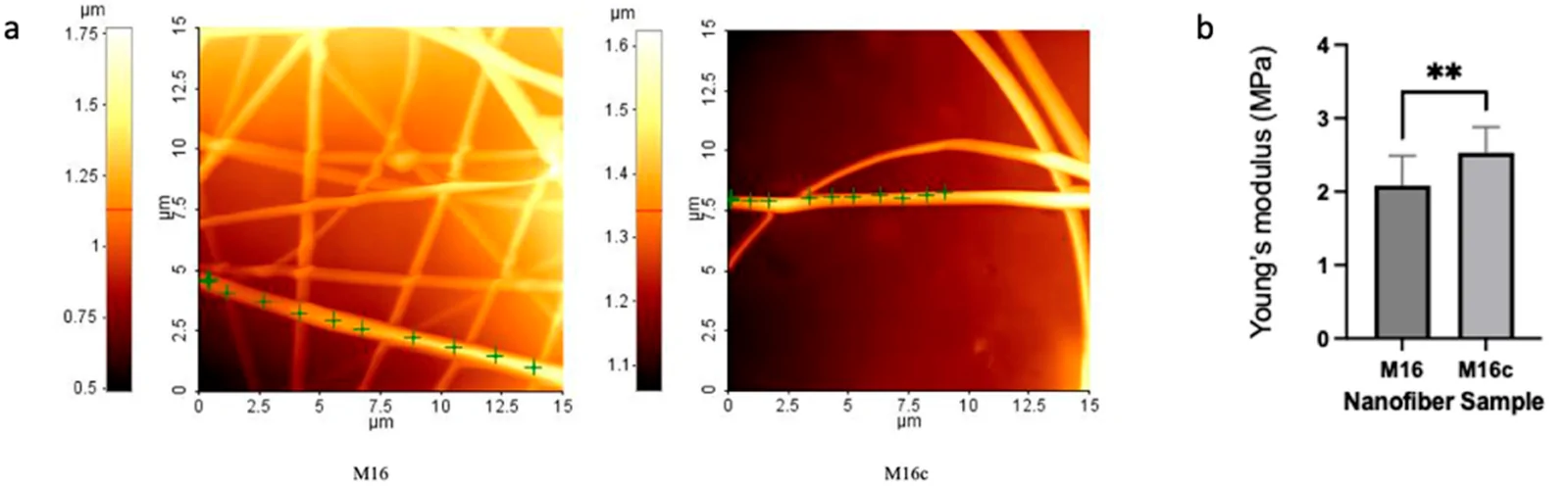
(**a**) Representative images of indentation regions for calculation of Young’s modulus where M16 is the raw fiber and M16c is the MSN-incorporated herbal-loaded nanofiber. The green symbol indicates the indentation point/region selected for Young’s modulus measurement. (**b**) AFM-based Young’s modulus of electrospun single fibers. Statistically significant differences were indicated (** *p* < 0.001).

The mechanical properties of the optimized nanofibers were subsequently evaluated by atomic force microscopy (AFM)-based nanoindentation (Figure 4). Representative indentation images demonstrated uniform deformation behavior across the analyzed fibers. The unloaded nanofibers (M16) exhibited a Young’s modulus of approximately 2.1 MPa, whereas incorporation of MSNs and HP extract (M16c) significantly increased the Young’s modulus to approximately 2.5 MPa (*p* < 0.001). The enhanced stiffness of the loaded nanofibers indicates that incorporating MSNs and HP improved the mechanical performance of the electrospun scaffold while preserving its homogeneous fibrous architecture distribution.

#### 2.2.3. FTIR Characterization of Optimized Nanofibers

**Figure 5:**
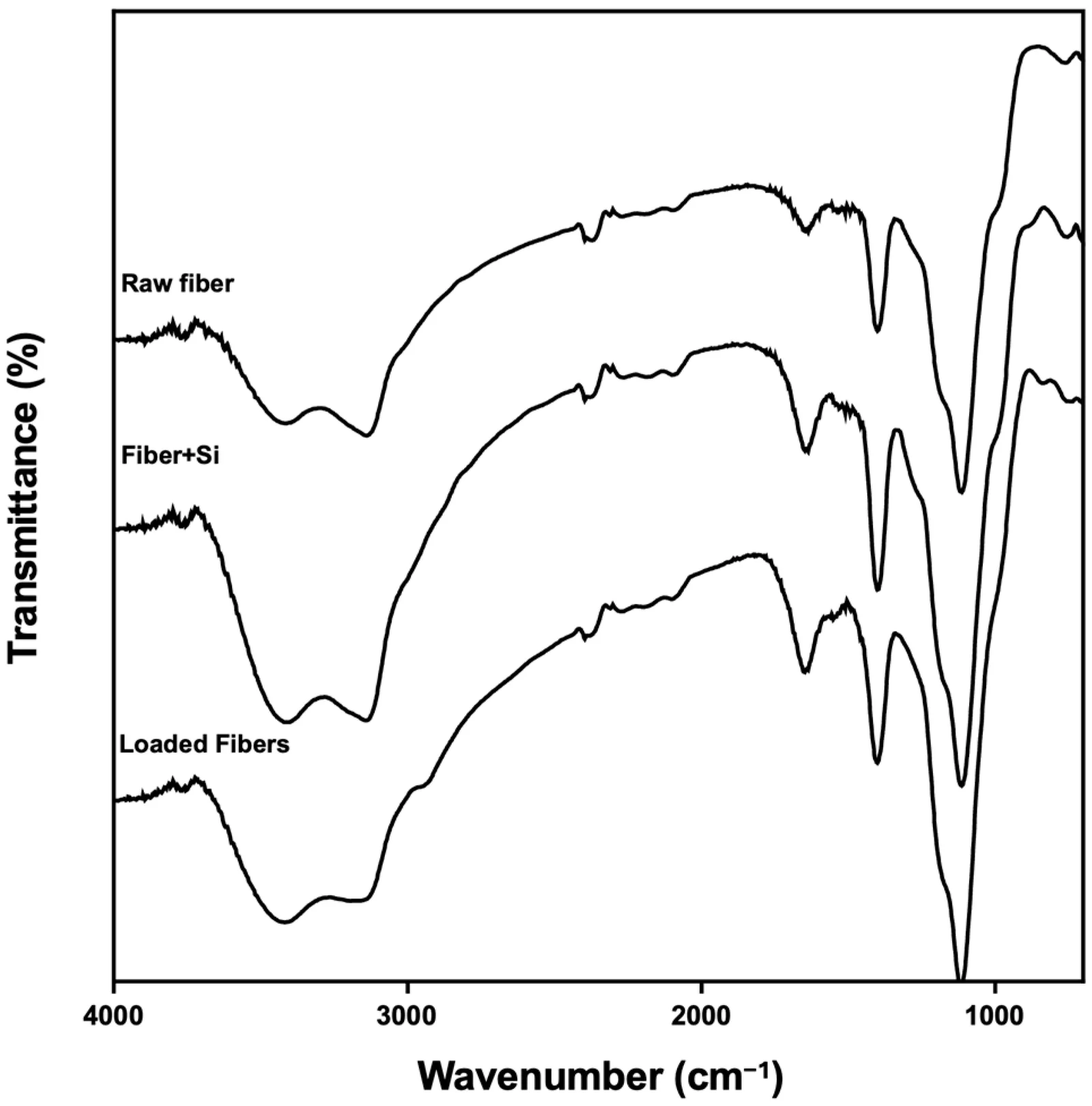
FTIR spectra of the raw nanofibers (M16), silica-containing nanofibers (Fiber + Si) (M16a), and MSN/HPE-loaded nanofibers (M16c).

Fourier Transform Infrared (FTIR) spectroscopy was employed to characterize the chemical structure of the electrospun nanofibers and to verify the successful incorporation of silica nanoparticles and *Hypericum perforatum* extract. The FTIR spectra of the raw nanofibers, silica-containing nanofibers (Fiber + Si), and *Hypericum perforatum*-loaded nanofibers are presented in Figure 5.

The FTIR spectrum of the raw electrospun nanofibers exhibited a broad absorption band centered at approximately 3400 cm^−1^, corresponding to the overlapping stretching vibrations of hydroxyl (–OH) and amine (–NH) groups originating from polyvinyl alcohol (PVA), chitosan, gelatin, and gum acacia. The broadness of this peak indicates extensive intermolecular hydrogen bonding within the polymeric matrix. The weak absorption band near 2930 cm^−1^ is assigned to the asymmetric and symmetric stretching vibrations of aliphatic –CH_2_ and –CH_3_ groups. The absorption band around 1640 cm^−1^ is attributed to the amide I (C=O stretching) vibration of gelatin together with the bending vibration of adsorbed water molecules, while the peak near 1550–1560 cm^−1^ corresponds to the amide II band arising from N–H bending and C–N stretching vibrations of chitosan and gelatin. The band observed around 1425–1430 cm^−1^ is associated with CH_2_ bending and O–H deformation vibrations, whereas the strong absorption between 1050 and 1100 cm^−1^ is assigned to C–O and C–O–C stretching vibrations of the polysaccharide backbone. These characteristic bands confirm the successful formation of the electrospun PVA/chitosan/gelatin/gum acacia composite nanofibers [24,25,26].

Following silica incorporation, additional characteristic silica bands were observed. The intense absorption band between 1060 and 1100 cm^−1^ corresponds to the asymmetric stretching vibration of the Si–O–Si network, while the peak around 800 cm^−1^ is assigned to the symmetric Si–O–Si stretching vibration. The broad absorption extending from 3200 to 3500 cm^−1^ is associated with hydrogen-bonded silanol (Si–OH) groups and adsorbed water molecules. The appearance and increased intensity of these characteristic bands confirm the successful incorporation of silica nanoparticles within the electrospun nanofibrous matrix [15,27].

After loading with *Hypericum perforatum* extract, the characteristic absorption bands of the polymeric matrix remained largely unchanged, indicating that the electrospinning and loading processes preserved the scaffold’s chemical integrity. Nevertheless, slight broadening of the hydroxyl stretching band around 3400 cm^−1^ and minor variations in the intensity of the bands located at 1600–1650 cm^−1^ and 1050–1100 cm^−1^ were observed [28]. These changes are attributed to hydrogen-bonding interactions between the hydroxyl-rich phytochemicals of *Hypericum perforatum*, including flavonoids, phenolic acids, hypericin derivatives, and hyperforin, and the hydroxyl and amino functional groups of the polymeric matrix. Because the extract consists primarily of naturally occurring polyphenolic compounds containing hydroxyl and carbonyl functional groups, its characteristic FTIR bands overlap with those of the polymers, resulting in peak broadening rather than the appearance of distinct new absorption bands.

### 2.3. Encapsulation Efficiency and In Vitro Hypericin Release

Hypericin encapsulation was quantified using a validated calibration curve with limits of detection and quantification of 1.3% and 4.4%, respectively. The encapsulation efficiency (EE%) ranged from 86% to 96% for all formulations, with no statistically significant difference between nanofibers containing MSNs and those without MSNs (Figure S4b).

**Figure 6:**
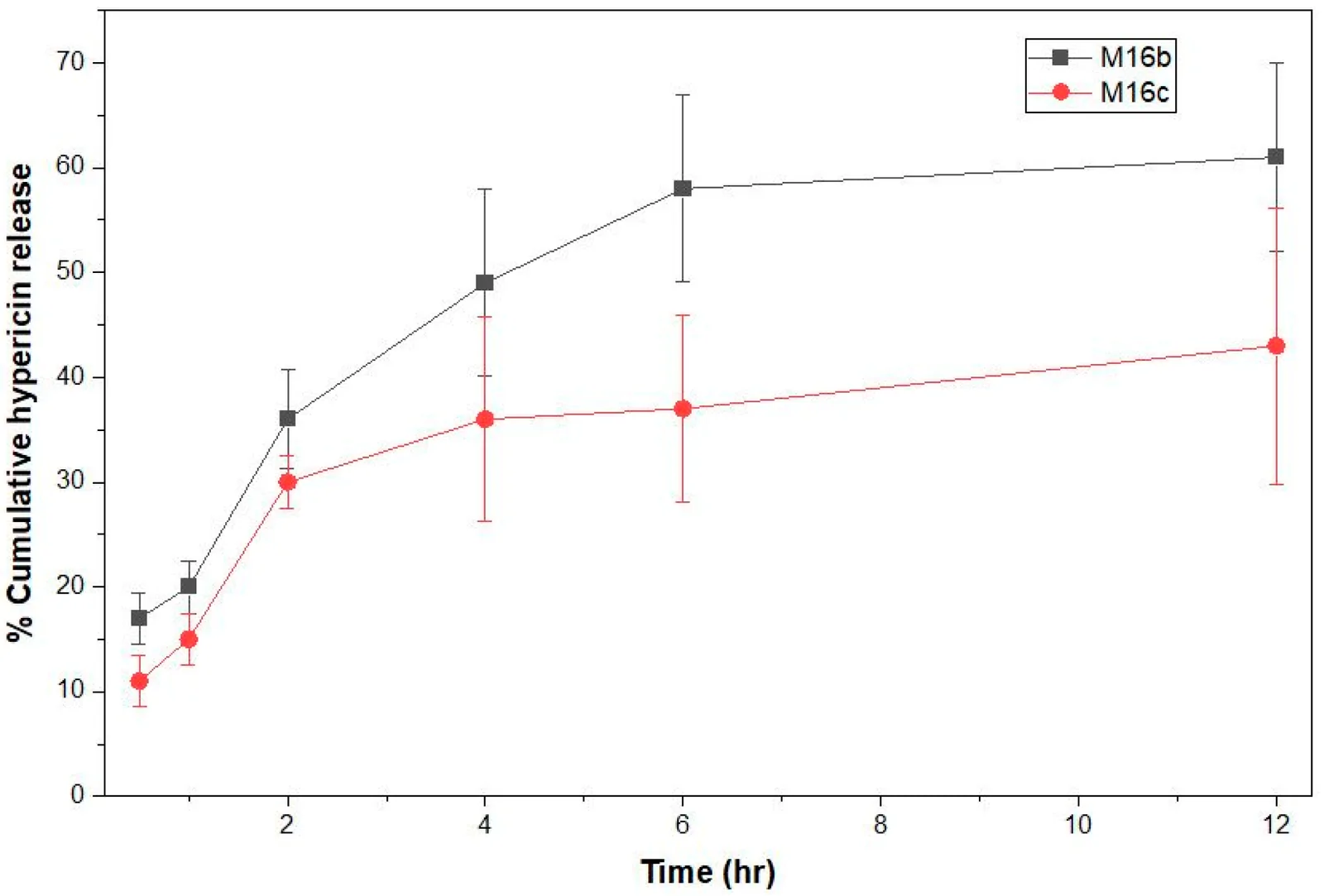
Cumulative hypericin release profiles from herbal-loaded nanofibers with and without MSNs.

The release profile of hypericin exhibited a biphasic pattern characterized by an initial burst release during the first 2 h followed by sustained release over 12 h (Figure 6). Incorporation of MSNs significantly delayed drug release. At 6 h, cumulative release reached approximately 37% for M16c compared with 58% for M16b. After 12 h, cumulative release increased to approximately 43% and 62%, respectively, demonstrating the ability of MSNs to prolong hypericin release.

### 2.4. Antimicrobial Activity

The antimicrobial activity of the developed nanofibers was evaluated against Gram-positive bacteria, Gram-negative bacteria, and *Candida albicans* (Table 1). The methanolic HPE produced inhibition zones ranging from 25 to 29 mm, corresponding to approximately 73% of the activity of the reference antimicrobial agents.

Herbal-loaded nanofibers also exhibited broad-spectrum antimicrobial activity, producing inhibition zones of 19–22 mm (approximately 55% efficacy). Incorporation of MSNs significantly enhanced antimicrobial performance, increasing inhibition zones to 23–30 mm against all tested microorganisms and improving the overall efficacy to approximately 70% of the positive controls. The highest inhibition zone (30 ± 0.5 mm) was observed against *C. albicans.*

### 2.5. Anti-Inflammatory Activity

**Figure 7:**
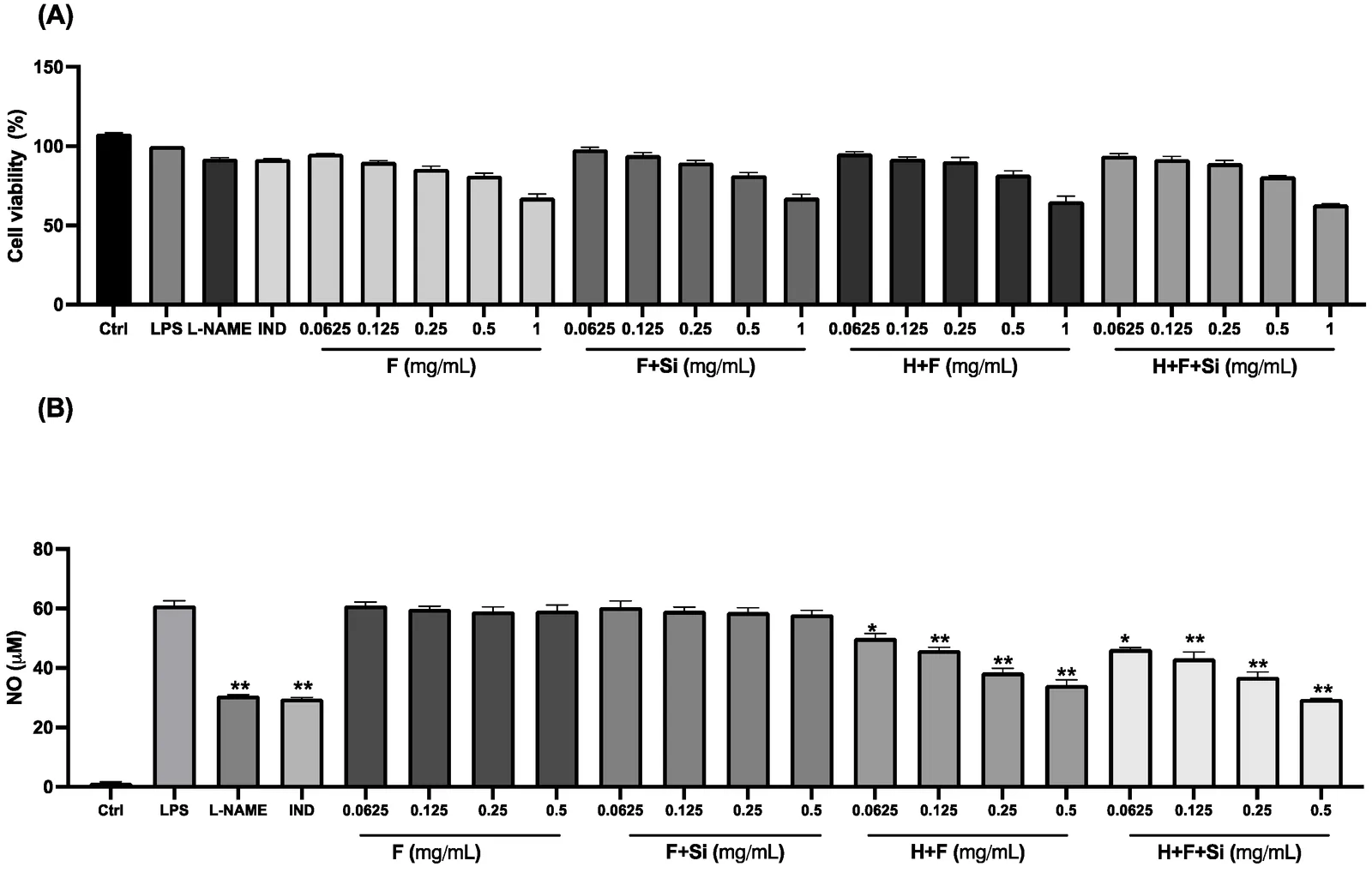
Effects of nanofiber mat extracts on RAW 264.7 cells: (**A**) cell viability and (**B**) nitrite production in LPS-stimulated cells. Ctrl: control group treated with DMEM; LPS: control group only stimulated with LPS; LPS: lipopolysaccharides from E. coli; L-NAME (100 µM): N(gamma)-nitro-L-arginine methyl ester (1+); IND: indomethacin (100 µM); F: raw nanofibers; F + Si: MSN-loaded nanofibers; F + H: herbal-loaded nanofibers; F + H + Si: MSN and herbal-loaded nanofibers. Statistically significant differences were indicated for each compound vs. LPS (* *p* < 0.05, ** *p* < 0.001). All experiments were performed in triplicate.

The cytocompatibility of the nanofiber extracts toward RAW 264.7 macrophages was first evaluated using the MTT assay to determine the concentrations suitable for anti-inflammatory assessment (Figure 7A). Cell viability remained above the ISO 10993-5 threshold (70%) at concentrations up to 0.5 mg mL^−1^ for all formulations, whereas exposure to 1 mg mL^−1^ significantly reduced cell viability [29]. Accordingly, concentrations ≤0.5 mg mL^−1^ were selected for subsequent anti-inflammatory evaluation. Only non-cytotoxic concentrations maintaining ≥70% cell viability was included in the anti-inflammatory assessment.

The anti-inflammatory activity of the nanofiber formulations was assessed by measuring nitric oxide (NO) production in lipopolysaccharide (LPS)-stimulated RAW 264.7 macrophages (Figure 7B). LPS stimulation markedly increased nitrite production to 60.99 ± 1.62 μM compared with the untreated control. Both HP-loaded nanofibers (F + H) and MSN-incorporated HP-loaded nanofibers (F + H + Si) significantly suppressed NO production in a concentration-dependent manner (*p* < 0.001). At the highest tested concentration, the F + H + Si formulation exhibited the greatest inhibitory effect, reducing nitrite production by approximately 51%, which was comparable to the positive controls, indomethacin and L-NAME. In contrast, the unloaded nanofibers (F) and MSN-loaded nanofibers without HP (F + Si) showed no significant inhibition of NO production.

### 2.6. In Vitro Cell Migration

**Figure 8:**
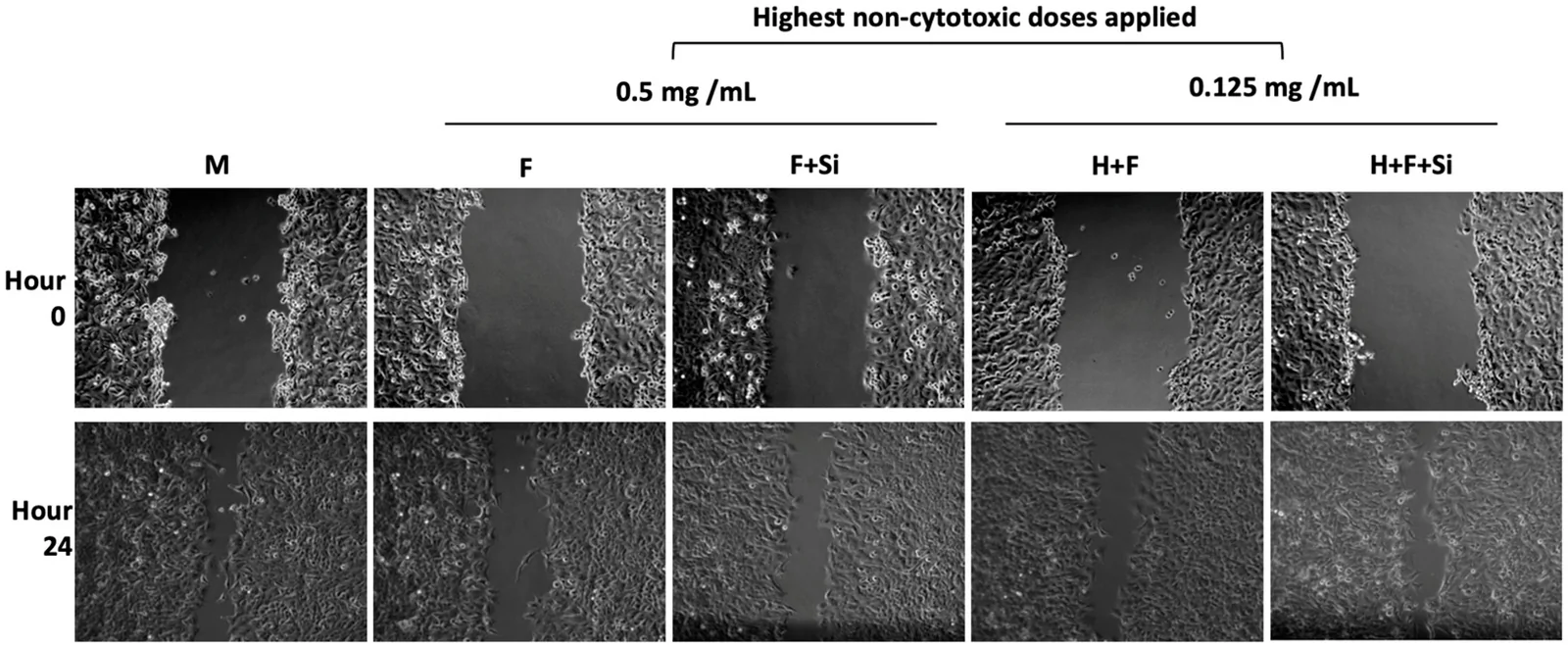
Effects of nanofiber mat extracts on cell migration. F: raw nanofibers, F + Si: MSN-loaded nanofibers, F + H: herbal-loaded nanofibers, F + H + Si: MSN and herbal-loaded nanofibers. All experiments were performed in triplicate.

The wound-healing potential of the developed nanofiber formulations was investigated using an in vitro scratch assay on L929 fibroblasts (Figure 8). Based on the cytocompatibility results, the two highest non-cytotoxic concentrations of each formulation were selected for evaluation. Cell migration was monitored over 24 h and compared with the untreated control.

The untreated control exhibited approximately 74% wound closure after 24 h. All tested formulations, regardless of concentration, exhibited cell migration rates comparable to the control; however, no statistically significant differences were observed relative to the control group. Thus, the incorporation of MSNs into herbal-loaded nanofibers did not significantly enhance wound closure under the experimental conditions.

### 2.7. Cytocompatibility and Genetic Safety

#### 2.7.1. Cytocompatibility

The cytocompatibility of the nanofiber formulations was further evaluated using L929 fibroblasts after 24 h of exposure (Figure S5). Cell viability remained above the ISO 10993-5 acceptance criterion (70%) at lower concentrations for all formulations [29]. Cytotoxic effects were observed at 1 mg mL^−1^ for the unloaded and HP-loaded nanofibers, whereas MSN-containing formulations exhibited reduced cell viability at concentrations of 0.5 and 1 mg mL^−1^.

#### 2.7.2. Mutagenicity Assessment

The mutagenic potential of the developed nanofibers was evaluated using the Ames test with *Salmonella typhimurium* TA98 and TA100 strains in the presence and absence of S9 metabolic activation (Table S2). None of the tested formulations produced a significant increase in the number of revertant colonies compared with the negative control, indicating the absence of detectable mutagenic activity under the experimental conditions.

#### 2.7.3. Genotoxicity Assessment

**Figure 9:**
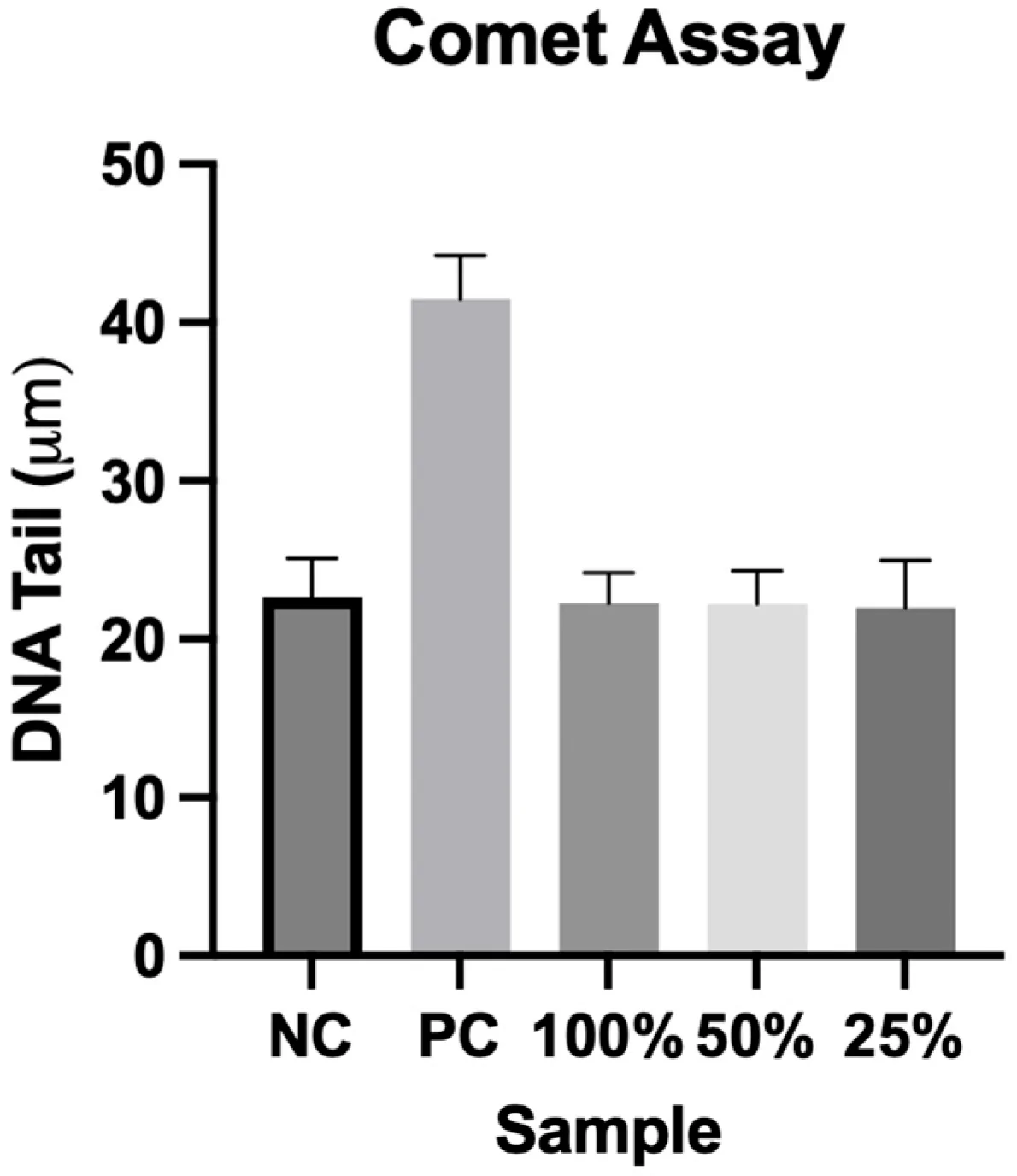
Quantification of comet extent by image analysis. NC: negative control, PC: positive control; different concentrations of herbal-loaded MSN-containing nanofiber mat were used (100%, 50%, 25%). Statistically significant differences were indicated between PC and the rest of the samples (*p* < 0.0001).

Genotoxicity was further assessed using the alkaline comet assay (Figure 9). The positive control exhibited extensive DNA migration, confirming the sensitivity of the assay, whereas treatment with MSN-incorporated HP-loaded nanofiber extracts at 25%, 50%, and 100% concentrations did not produce significant DNA damage compared with the negative control (*p* < 0.0001). These findings indicate that the developed nanofiber formulations exhibited no detectable genotoxic effects under the tested conditions.

## 3. Discussion

This study developed MSN herbal-loaded nanofibers using electrospinning, a widely used technique for producing nanofibrous mats with high surface area and porosity, features ideal for wound dressings. The polymeric precursor solution included chitosan, polyvinyl alcohol, herbal extracts (acacia gum and *H. perforatum*) and mesoporous silica nanoparticles. High-voltage application generated uniform nanofibers, enhancing drug-loading capacity and enabling controlled bioactive release. Optimization of polymer composition and electrospinning parameters led to a refined formulation. SEM analysis confirmed uniform nanofiber morphology and successful MSN incorporation, contributing to the mesoporous structure and improved drug delivery properties. These results are consistent with previous reports showing that lower CS content in PVA/CS/gelatine blends reduced bead formation and improved fiber uniformity. For instance, decreasing the chitosan ratio in the CS/PVA mixture from 50:50 to 30:70 eliminated beading [30]. Also, our results revealed that addition of gelatine to the blends prepared in 90% acetic acid led to smoother nanofibers with fewer beads [31]. Our findings also align with studies showing that higher polymer concentrations enhance fiber formation. A fixed PVA:CS ratio (80:20) in 2% acetic acid exhibited improved morphology as precursor concentration increased from 3% to 5 wt% [25]. In another study, blending 8% PVA with 2% CS and 10% gelatine produced fibers with minor bead formation and increasing fiber diameter proportional to PVA content [7], consistent with our data. Acacia gum was also effective in improving nanofiber properties. A 10% PVA and 1% acacia gum solution (5:1 ratio) enhanced fiber formation and conferred antifungal and wound healing properties [32,33]. The gum acted as a thickening agent and contributed biological benefits [9]. These results validate the expected performance and characteristics of the material, further supporting its potential application. The nanoindentation test results revealed a significant increase in Young’s modulus in the MSN-containing herbal-loaded nanofiber mat compared to the raw nanofiber mat without any additives. This enhancement highlights the beneficial effects of HPE and MSNs on the mechanical stability of the mats [34]. These findings suggest that the MSN-incorporated herbal-loaded nanofiber mat is more robust, which may support its application in biomedical fields requiring durable and flexible materials. Typically cylindrical, MSNs range from 50 to 500 nm in diameter with pore sizes between 2 and 20 nm, functioning as efficient carriers and reservoirs in drug delivery, adsorption, and heterogeneous catalysis applications [35]. Particle size analysis in our study revealed two distinct ranges—150–190 nm and 500–530 nm—for the MSNs used, both within the expected and safe size range for biomedical nanoparticles. Zeta potential analysis yielded an average value of −24.1 mV, confirming successful carboxylate group functionalization on the MSN surfaces [13]. SEM and TEM images confirmed cylindrical morphology, resembling silica from plant sources such as rice husk and sugarcane bagasse. These results highlight the potential of MSNs as effective carriers for therapeutic agents [35].

FT-IR findings demonstrate that the integration of herbal extracts and functionalized MSNs into chitosan-based electrospun nanofibers results in a composite material with enhanced physicochemical, mechanical, and potentially therapeutic properties. This positions the developed nanofiber mats as promising candidates for advanced wound care applications, combining structural integrity with bioactivity. Importantly, no additional absorption bands corresponding to newly formed chemical bonds were detected after drug loading. This observation indicates that HPE was physically entrapped within the electrospun nanofibers via hydrogen bonding and other intermolecular interactions rather than through covalent chemical reactions. Such physical encapsulation is advantageous because it preserves the bioactive constituents of the herbal extract while maintaining the structural integrity of the nanofibrous scaffold. Overall, the FTIR results confirm the successful fabrication of PVA/CS/G/AG electrospun nanofibers, the effective incorporation of silica nanoparticles, and the successful encapsulation of *Hypericum perforatum* extract. The absence of new chemical bonds, together with the observed peak broadening and slight shifts, suggests good compatibility among the polymers, silica nanoparticles, and the herbal extract, resulting in a stable composite nanofibrous system suitable for biomedical applications.

Several studies have examined H. perforatum extracts for wound healing. Hypericin, the chromophore responsible for the reddish color, is typically confirmed by spectrophotometry, showing an absorption peak near 590 nm, consistent with literature values. Prior work often used HP oil, limiting precise EE% and release assessment. In one study, HP-loaded chitosan nanoparticles in an agarose film demonstrated 90% EE and an 82% release after 4 h in PBS [36]. In our study, the EE% ranged from 86% to 96%, and the release profile showed a burst in the first 2 h (30% for herbal-loaded and 36% for MSN herbal-loaded nanofibers), followed by a sustained release over 12 h (62% and 43%, respectively). These findings suggest a prolonged therapeutic action of the formulation, supporting its application in wound healing systems.

Antimicrobial activity of HP-loaded nanofibers is consistent with the known properties of H. perforatum, which contains a range of active compounds including polysaccharides and naphthodianthrones. The enhancement observed with MSN incorporation is likely due to the controlled, sustained release of herbal actives, thereby allowing prolonged antimicrobial effects. Variability in antimicrobial activity across formulations may stem from differences in the physicochemical properties of the nanofibers and the efficiency of herbal compound encapsulation. The improved performance of MSN-loaded formulations suggests a synergistic effect between MSNs and the plant extract, possibly by increasing surface interaction with microbial cells or enhancing stability of active compounds. These findings are supported by previous studies. For instance, gelatine nanofibers loaded with HP oil showed inhibition zones of 7.83 mm (*E. coli)* and 17 mm (*S. aureus*), indicating potential for wound care [37]. Likewise, HP oil-loaded chitosan cryogels demonstrated antibacterial activity against *E. coli* (14 mm), *L. pneumophila* (13 mm), *P. aeruginosa* (12 mm), *S. aureus* (11 mm), and additional pathogens like *E. hirae*, *B. cereus*, and *C. albicans* (10 mm each) [38], supporting its suitability for tissue engineering and wound healing applications.

Beyond antimicrobial effects, anti-inflammatory activity is critical for wound repair. HP extracts have shown >30% inhibition of PGE2 and NO in RAW 264.7 macrophages [21]. In this study, MSN-containing herbal-loaded nanofibers achieved approximately 51% nitrite inhibition at the highest tested concentration, comparable to L-NAME and indomethacin. This enhanced response likely reflects MSN-mediated control of release and improved bioactivity, underscoring the strong anti-inflammatory potential of MSN–HP nanofibers for wound care. The enhanced bioactivity and sustained release observed in MSN-incorporated nanofibers are likely driven by favorable interactions between hypericin and the mesoporous silica matrix. Hypericin contains multiple phenolic hydroxyl groups that can form hydrogen bonds with surface silanol (Si–OH) groups of mesoporous silica nanoparticles, promoting adsorption within the mesopores and limiting π–π stacking-induced aggregation. Confinement within the porous structure may also protect hypericin from oxidative and photodegradative degradation, thereby preserving its biological activity. In addition, adsorption-driven diffusion from the negatively charged silica surface contributes to prolonged release kinetics. Beyond their carrier role, MSNs have been reported to modulate local redox and inflammatory pathways, which may further enhance hypericin-mediated suppression of nitric oxide production [14,39].

Cell migration assays model early wound repair and reveal how cells respond to biomaterials. Prior work with CS/collagen films loaded with allantoin and lidocaine hydrochloride enhanced gap closure at 6 and 24 h in mesenchymal stem cells [40]. Here, fibroblast migration was evaluated using L929 cells, and wound closure was assessed by comparing images taken at 0 and 24 h after treatment with MSN herbal-loaded nanofibers at different concentrations. Despite the notable anti-inflammatory activity observed in these formulations, the results revealed no significant improvement in cell migration compared to the control group. This likely reflects limited assay sensitivity and/or suboptimal dosing and/or time points, underscoring the need for in vivo studies to better assess regenerative potential.

The results inform both biological activity and safety. In L929 cells, cytotoxicity appeared at relatively low concentrations—0.5 mg/mL for raw and MSN-containing nanofibers, and 0.25 mg/mL for MSN-containing herbal-loaded nanofibers—highlighting the need for dose optimization. This contrasts with reports of no cytotoxicity for HP-loaded systems after 24 h MTT [36,37], suggesting a dose-dependent effect in our formulations and the importance of balancing efficacy with safety.

While previous studies have primarily focused on the cytotoxicity of nanofiber-based wound dressings, this study offers a more comprehensive safety evaluation by incorporating both mutagenicity and genotoxicity assessments. The tested MSN-containing herbal-loaded nanofiber mats were evaluated using the Ames test (TA98 and TA100 strains, with and without S9 metabolic activation) and the comet assay. Results showed no significant increase in revertant colonies or DNA damage, indicating that the nanofiber mats are non-mutagenic and non-genotoxic. These findings suggest a favourable genetic safety profile, essential for materials intended for wound healing and tissue regeneration. The study underscores the need for rigorous safety evaluations that extend beyond basic cytotoxicity to ensure the safe therapeutic application of nanomaterial.

## 4. Materials and Methods

### 4.1. Material

CS, PVA, and G were obtained from Merck Chemicals (Darmstadt, Germany). St. John’s Wort extract capsule food supplements (Solgar, Leonia, NJ, USA) and olive oil extract (Hünnap, Istanbul, Türkiye) were purchased from a local pharmacy in Istanbul, Türkiye. AG, which is dried gummy exudates of acacia, was obtained from a herbal medical store. Acetic acid, methanol, and other reagents were obtained from Sigma-Aldrich Chemie GmbH, Steinheim, Germany. Dulbecco’s phosphate-buffered saline (DPBS; Gibco, Life Technologies Limited, Paisley, UK) was used. Salmonella typhimurium strains, S9 mix, and nutrient broth were obtained from Molecular Toxicology, Inc. (MOLTOX; Boone, NC, USA).

### 4.2. Synthesis of MSNs

The MSNs used in this study were previously synthesized and characterized at Tabriz University of Medical Sciences, Iran, according to the method reported by Porrang et al. [23]. In this approach, silica obtained from agricultural waste, including rice and wheat husks, was used as a biogenic precursor for the preparation of mesoporous silica nanoparticles. The use of renewable agricultural waste as the silica source reduces dependence on conventional synthetic silica precursors and forms the basis for describing the process as a green synthesis approach. The previously prepared MSN samples were used in the present study without further modification.

The mesoporous characteristics of the MSNs were established in the original characterization study [23]. The nanoparticles exhibited a Brunauer–Emmett–Teller (BET) specific surface area of approximately 650 m^2^/g, a total pore volume of 0.85 cm^3^/g, and an average pore diameter of approximately 5.2 nm. The pore size distribution was mainly within the 3–8 nm range, consistent with the IUPAC definition of mesoporous materials. In addition, the nitrogen adsorption–desorption isotherm showed a characteristic Type IV profile with an H1-type hysteresis loop, further supporting the mesoporous structure of the nanoparticles [23].

### 4.3. Preparation of Electrospinning Solutions

CS (medium molecular weight), PVA (MW ~85,000, 88% hydrolyzed), G (Type B), acetic acid (80% *v*/*v*) and deionized water were used for the preparation of electrospinning solutions. CS was dissolved at concentrations ranging from 1% to 3% (*w*/*v*) in 80% (*v*/*v*) acetic acid under continuous stirring at room temperature for 4–6 h until a clear and homogeneous solution was obtained. Separately, PVA was dissolved in deionized water at 80–90 °C under constant stirring for 2–4 h to obtain a clear solution. PVA solutions were prepared at varying concentrations (5–12% *w*/*v*). Gelatin was dissolved in deionized water at 40–50 °C with continuous stirring until fully solubilized to prepare a 10% (*w*/*v*) G solution. The PVA solution was then added dropwise into the CS solution under continuous stirring, followed by the gradual addition of the gelatin solution. The final blend was stirred at room temperature for an additional 2–4 h to ensure homogeneity. Prior to electrospinning, the prepared solution was degassed using ultrasonication or vacuum treatment to eliminate entrapped air bubbles.

Detailed preparation ratios, active agent incorporation strategies, and polymer blend compositions are provided in the Supplementary Data (Table S1).

### 4.4. Electrospinning

To optimize nanofiber formation, various preparation methods and polymer blend ratios were systematically evaluated. Electrospinning was performed using a conventional laboratory-scale electrospinning system (Inovenso NE-300; Istanbul, Türkiye) equipped with a stationary collector.

A blunt-ended metallic needle was employed and the collector was positioned at a fixed distance from the needle tip. The polymer solution was delivered at a controlled flow rate using a syringe pump (NE-300 Just Infusion™ Syringe Pump, New Era Pump Systems, Inc., Farmingdale, NY, USA) to ensure consistent fiber formation. Critical electrospinning parameters, including flow rate, applied voltage, and needle-to-collector distance, were optimized through iterative experiments. Detailed values for each parameter are provided in the Supplementary Data (Table S1). Environmental conditions, such as ambient temperature and relative humidity, were maintained within controlled ranges (22 °C ± 2 °C, 50% RH ± 3%) using the facility’s air conditioning system to ensure process reproducibility and stability throughout the electrospinning procedure.

### 4.5. Characterization of Nanofibers

The morphology of nanofibers and MSNs was analyzed using scanning electron microscopy (SEM; Zeiss EVO 40,Heidelberg Germany) and transmission electron microscopy (TEM, JEM-2100Plus, JEOL, Tokyo, Japan). Samples were coated with a thin gold/palladium layer using a Leica EM ACE200 sputter coater. MSN powders were mounted on carbon tape for SEM, while for TEM, MSNs were dispersed in ethanol and applied to grids. Imaging was performed at 200 kV using a LaB6 electron source. Fourier transform infrared spectroscopy (FTIR; PerkinElmer, Waltham, MA, USA) with the KBr method was performed to identify functional groups in nanofibers and MSNs over a range of 4000–400 cm^−1^. Atomic force microscopy (AFM; Park Systems XE 100, Gwacheon-si, Republic of Korea) provided surface topography and mechanical data, analyzed via XEI software. Mechanical properties, including Young’s modulus, were assessed via nanoindentation. Nanofibers collected on glass slides were measured at ten random points on twelve fibers per sample. Young’s modulus was calculated using the Hertz model, appropriate for polymer composites [34]. Dynamic light scattering (DLS; Malvern Zetasizer NanoZS, Malvern Panalytical, Malvern, UK) determined MSN particle size and zeta potential. A refractive index of 1.42 was used, and measurements were performed in distilled water and repeated 4 times for accuracy. Fiber diameters were measured using the ImageJ software (Version 1.54). At least 100 fibers were measured to calculate fiber diameters from the SEM data.

### 4.6. Characterization and Quantification of Hypericum Perforatum Extracts

The identification and quantification of HPE were performed using hypericin as a reference. UV-Vis spectrophotometry (Evolution 300, Thermo Fisher Scientific, Madison, WI, USA) was conducted over 400–700 nm, with hypericin showing peak absorbance at 580–600 nm. Both oil and powdered capsule extracts (sourced locally) were analyzed. Acetone served as the solvent for the oily extract, while methanol and PBS were used for the powdered extract. Samples were sonicated for 30 min and filtered before analysis (Figure S4c) [41]. After several trials, powdered capsule extracts dissolved in methanol were selected for the following experiments. For extract preparation, 100 mg of HP powder was dissolved in 10 mL methanol, sonicated (30 min), and left to settle for 24 h. The supernatant was centrifuged and evaporated at 50 °C. For quantification, the residue was reconstituted in 10 mL of 80% acetic acid. After 10 min of magnetic stirring, dilutions were prepared (100%, 75%, 50%, 25%, 10%, and 5%), hypericin content was measured at 588 nm, and a calibration curve was plotted (Figure S4a) [36,42]. All measurements were performed in triplicate to ensure reproducibility. This methodology ensured accurate hypericin quantification and the reliability of HP extracts for nanofiber formulation.

### 4.7. Encapsulation Efficiency and Release Studies

To incorporate HPE into the nanofiber, HPE residue was reconstituted with 10 mL of the polymer mixture and stirred for at least 10 min before degassing and electrospinning. Encapsulation efficiency (EE%) was determined from 1 mL of polymer mixture, yielding ~120 mg of nanofibers. Fibers were dissolved in 4 mL of 80% acetic acid at 80 °C in a shaking water bath. After centrifugation, the supernatant was analyzed via UV-Vis spectrophotometry at 588 nm. The procedure was repeated in triplicate for reproducibility. EE% was calculated using the formula [36]:

EE% = ((Weight of drug in nanoparticles)/(Weight of feeding drugs)) × 100

For release studies, 120 mg of nanofibers was incubated in 4 mL phosphate buffer (pH 7.4) at 37 °C with a shaking water bath (WiseBath WSB-30, DAIHAN Scientific Co., Ltd., Wonju-si, Gangwon-do, Republic of Korea). Samples were collected over 12 h and analyzed spectrophotometrically at 588 nm to determine cumulative release profiles [42]. The hypericin release studies were conducted both from nanofiber samples with and without MSNs. Samples without herbal extracts were used as blanks during measurements.

### 4.8. Antimicrobial Activity

Antimicrobial activity was assessed via the disk diffusion method against *Enterococcus hirae* (ATCC 10541), *Enterococcus faecalis* (ATCC 29212), *Staphylococcus aureus* (ATCC 6538), *Escherichia coli* (ATCC 10536), *Acinetobacter baumannii* (ATCC 19606), *Pseudomonas aeruginosa* (ATCC 15442), and *Candida albicans* (ATCC 10231). Inhibition zones were measured to determine efficacy. Ofloxacin (5 µg) and nystatin (100 U) served as positive controls [38]. Ofloxacin standard disks (5 μg) were employed as positive controls and reference standards for the agar disk diffusion assay. Bacterial and fungal inocula were prepared and adjusted to a 0.5 McFarland turbidity standard before being evenly spread onto Mueller–Hinton Agar for bacterial strains and Sabouraud 2% Dextrose Agar for fungal strains using sterile cotton swabs. Sterile blank paper disks (6.0 mm in diameter) were loaded with the respective test formulations and subsequently positioned onto the inoculated agar surfaces. Following incubation, inhibition zones surrounding the disks were evaluated after 18–24 h at 37 °C for bacterial cultures and 24–72 h at 25 °C for fungal cultures. Antimicrobial efficacy was assessed based on the diameter of the clear inhibition zones formed around the test disks.

### 4.9. In Vitro Anti-Inflammatory Effect and Cell Migration

Cell lines and culture conditions: RAW 264.7 mouse macrophages (ATCC^®^ TIB-71™) and L929 fibroblast cells (ATCC, Manassas, VA, USA) were cultured in DMEM supplemented with 10% fetal bovine serum and 1% penicillin–streptomycin. Cells were maintained at 37 °C in a 5% CO_2_ atmosphere.

#### 4.9.1. Sample Preparation

Scaffolds were cut and extracted in culture medium (6 cm^2^/mL) per the ISO 10993-12:2012 guideline [43]. Samples were incubated at 37 °C and 5% CO_2_ for 24 h, then diluted in DMEM for assays.

#### 4.9.2. Anti-Inflammatory Activity

Nitric oxide (NO) inhibition was assessed via nitrite quantification using the Griess reagent. RAW 264.7 cells (5 × 10^4^/well) were seeded in 96-well plates and incubated 24 h. Cells were pre-treated with scaffold extracts (0.0625–0.5 mg/mL) for 2 h, then stimulated with lipopolysaccharide (LPS, 1 µg/mL) for 22 h. Supernatants were reacted with Griess reagent and absorbance at 540 nm was measured (Thermo Fisher Scientific, USA). Nitrite concentrations were calculated using a sodium nitrite standard curve. Indomethacin and L-NAME (100 mM) served as positive controls [44].

#### 4.9.3. Cell Migration Assay

Cell migration, essential for wound healing, was assessed using a scratch assay. L929 cells (1.5 × 10^5^ cells/mL) were seeded in 24-well plates and incubated at 37 °C and 5% CO_2_ until confluent. Pre-marked plates guided image alignment. After 24 h, a scratch was made using a cell scraper to simulate injury. Wells were rinsed with PBS and 500 µL of fresh media was added. Scaffold extracts (0.0625–0.5 mg/mL) were applied and DMEM served as control. Images were taken at 0 and 24 h using a 10× objective (Zeiss AxioCam, Oberkochen, Germany). Wound closure was analyzed with ImageJ (NCBI, USA) and expressed as migration area (%) [45]:

Gap closure% = [((Gap area at t_0_) − (Gap area at t_final_)/Gap area at t_final_)] × 100

### 4.10. Cytotoxicity Assay

Cytotoxicity of herbal extract-loaded nanofibers was assessed on RAW 264.7 macrophages and L929 fibroblasts using the MTT assay. RAW 264.7 cells were seeded at 5 × 10^4^ cells/well and L929 cells at 1 × 10^5^ cells/well in 96-well plates with DMEM containing 10% FBS and 1% penicillin–streptomycin. After 24 h incubation at 37 °C and 5% CO_2_, cells were treated with nanofiber extracts at 0.0625, 0.125, 0.25, and 0.5 mg/mL for 24 h. Then, 100 µL of 0.5 mg/mL MTT solution was added per well and incubated for 2 h. The MTT was removed, and 100 µL isopropanol added to dissolve formazan crystals. Absorbance was measured at 570 nm. Cell viability below 70% compared to untreated controls was considered cytotoxic. Viability (%) was calculated using the formula [45]:

Cell viability (%) = OD_570_ (sample) × 100/OD_570_ (medium control)

(OD: Optical Density)

### 4.11. Ames Test

The Ames test detects DNA-mutagenic substances using specific bacterial strains. The plate incorporation method was used to assess mutagenicity. Test tubes containing 2 mL of top agar with histidine–biotin solution were pre-warmed at 45 °C. Each tube was mixed with 0.05 mL test substance and 0.1 mL of a 12–14-h bacterial culture, vortexed, then poured onto minimal glucose agar plates. After 15 min solidification, plates were inverted and incubated at 37 °C for 48–72 h, and His^+^ revertant colonies were manually counted.

Metabolic activation was tested using S9 fraction (rat liver extract). For S9 assays, 0.5 mL fresh S9 mix was added before plating; S9-free assays excluded this mix. Positive controls for S9 assays were 2-aminofluorene (2-AF) for TA98 and TA100 strains. For S9-free assays, direct mutagens 4-nitro-o-phenylenediamine (NPD) and sodium azide (SA) were positive controls for TA98 and TA100, respectively. Plates were incubated at 37 °C for 48 h, and then colonies were counted.

The mutagenic index (MI) was calculated as:

MI = (mean revertant colonies in treated sample)/(mean revertant colonies in negative control).

A compound is considered mutagenic if a dose-dependent increase occurs and MI ≥ 2 at any concentration [46].

### 4.12. Comet Assay

L929 cells were seeded in 6-well plates (3 × 10^5^ cells/well) and incubated overnight. Cells were then treated with nanofiber extracts at 100%, 50% and 25% concentrations for 4 h. After treatment, cells were harvested, centrifuged at 1000 rpm for 5 min and resuspended in remaining medium. A 20 µL aliquot was mixed with 180 µL of pre-warmed low-melting-point agarose and then 45 µL of this mixture was spread on slides precoated with high-melting-point agarose. After setting at room temperature for 3 min, coverslips were removed and slides were placed in cold lysis solution at 4 °C in the dark. Slides were equilibrated in cold electrophoresis buffer for 20 min, then electrophoresed at 25 V and 300 mA for 20 min. Following electrophoresis, slides were rinsed with distilled water, immersed in Tris buffer for 5 min, fixed in ice-cold methanol (−20 °C) for 5 min and air-dried. DNA was stained with 20 µL Gel Red and covered with a coverslip. DNA damage was assessed using the BAB Bs200ProP Image System under fluorescence microscopy. Results were expressed as % DNA in the tail, analyzing 100 cells (50 per slide) [47].

### 4.13. Statistical Analysis

All experiments were performed in triplicate. Data were analyzed using one-way ANOVA followed by Tukey’s post hoc test with GraphPad Prism 9 (version 10.2.3).

## 5. Conclusions

In this study, a multifunctional electrospun nanofibrous scaffold incorporating green-synthesized mesoporous silica nanoparticles (MSNs) and *Hypericum perforatum* (HP) extract was successfully developed as a sustainable platform for wound-healing applications. The optimized PVA/chitosan/gelatin/gum acacia formulation produced uniform, bead-free nanofibers with improved mechanical properties while maintaining the structural integrity of both the polymeric matrix and the incorporated bioactive components. The green-synthesized MSNs exhibited suitable physicochemical characteristics for drug delivery and were effectively integrated into the electrospun scaffold without compromising fiber morphology or drug encapsulation.

The developed nanofibers demonstrated high encapsulation efficiency and a biphasic release profile, with MSN incorporation significantly prolonging hypericin release. This sustained release was accompanied by enhanced antimicrobial activity against Gram-positive bacteria, Gram-negative bacteria, and *Candida albicans*, together with significant inhibition of nitric oxide production in activated macrophages, highlighting the potential of the scaffold to simultaneously control microbial infection and modulate inflammation. Although the formulations did not significantly enhance fibroblast migration under the experimental conditions, they exhibited acceptable cytocompatibility at therapeutically relevant concentrations and showed no evidence of mutagenic or genotoxic effects.

Overall, the combination of green-synthesized MSNs with HP-loaded electrospun nanofibers provides a multifunctional and environmentally sustainable wound dressing with controlled drug-release capability, favorable biological activity, and an encouraging safety profile. Future studies should evaluate the long-term stability, biodegradation behavior, and therapeutic efficacy of the developed scaffold in relevant in vivo wound models to further establish its potential for clinical translation.

## Figures and Tables

**Table 1:** Antimicrobial Activities of Compounds (Zone of Inhibition/diameter in mm).

Compound	*E. hirae*	*E. faecalis*	*S. aureus*	*E. coli*	*A. baumannii*	*P. aeruginosa*	*C. Albicans*	% *
M16b	20 ± 1.2	21 ± 1.2	19 ± 0.3	21 ± 1.2	22 ± 0.8	21 ± 1.5	22 ± 0.2	55
M16c	29 ± 0.5	28 ± 0.9	24 ± 0.6	23 ± 1.2	25 ± 1.6	27 ± 1.0	30 ± 0.5	70
HPE	27± 0.6	28 ± 0.9	25 ± 0.6	27 ± 1.2	28 ± 1.2	29 ± 0.3	28 ± 0.4	73
Nystatin (100U)	-	-	-	-	-	-	38 ± 1.3	100
Ofloxacin (5 µg)	35 ± 0.5	39 ± 0.2	38 ± 1.2	37 ± 1.5	39 ± 0.3	39 ± 1.2	-	100

* Average efficacy % compared to control. M16b: HPE-loaded nanofibers; M16c: MSN/HPE-loaded nanofibers; HPE: *Hypericum perforatum* methanolic extract.

## Data Availability

The data used to support the findings of the present study are available from the corresponding authors upon request.

## Supplementary Materials

The following supporting information can be downloaded at: https://www.mdpi.com/article/10.3390/molecules31183257/s1, Table S1: The different polymer blends’ preparation ratios, methods, active agents added and electrospinning parameters; Table S2: Mutagenicity of the sample in *Salmonella typhimurium* TA98 and TA100 strains.; Figure S1: (a) Particle size intensity of MSNs, (b) zeta measurement results, and (c) FTIR spectrum of MSNs; Figure S2: Representative SEM images at 5K× of different formulations and the corresponding physical morphology; Figure S3: Fiber diameter distributions were determined from the SEM images using ImageJ for all M16 formulations; Figure S4: Hypericin absorbance spectrum and encapsulation efficiency studies. (a) Hypericin calibration curve after sample preparation (b) Actual calculated hypericin content. * *p* < 0.0001 HP and HP + MNNs compare to the RF: raw fiber. (c) Hypericin absorbance spectrum (P: extract powders in methanol (P-MeOH) and buffer (P-B) and extract oil in acetone (O-Acetone)); Figure S5: Cell viability results on L929 cell lines.

## Abbreviations

The following abbreviations are used in this manuscript:

2-AF	2-Aminofluorene
AFM	Atomic Force Microscopy
AG	Acacia Gum
ARE	Antioxidant/Electrophile Response Element
ASTM D 638	American Society for Testing and Materials standard for tensile properties of plastics
ATCC^®^	American Type Culture Collection
CS	Chitosan
Ctrl	Control group
DLS	Dynamic Light Scattering
DMEM	Dulbecco’s Modified Eagle Medium
DMSO	Dimethyl Sulfoxide
ELISA	Enzyme-Linked Immunosorbent Assay
F	Raw fiber
F + H	Raw fiber with herbal extract
F + H + Si	Raw fiber with herbal extract and MSNs
F +Si	Raw fiber with MSNs
FTIR	Fourier-transform Infrared Spectroscopy
G	Gelatin
HPE	*Hypericum perforatum* extract
IND	Indomethacin
KCl	Potassium Chloride
L-NAME	N(gamma)-nitro-L-arginine methyl ester (1+)
LOD	Limit of Detection
LOQ	Limit of Quantification
LPS	Lipopolysaccharide
MGA	Minimal Glucose Agar
MSNs	Mesoporous Silica Nanoparticles
MTT	3-(4,5-Dimethylthiazol-2-yl)-2,5-Diphenyltetrazolium Bromide
NTA	Nanoparticle Tracking Analysis
NO	Nitric oxide
NP	4-Nitro-o-phenylenediamine
PVA	Polyvinyl Alcohol
PBS	Phosphate-buffered saline
Pen-Strep	Penicillin–Streptomycin
RS comet assay	Reconstructed Skin Comet Assay
RSMN	Reconstructed Skin Micronucleus Test
RPM	Revolutions Per Minute
SA	Sodium Azide
SEM	Scanning Electron Microscopy
S9	A rat liver extract containing active liver enzymes
SPM	Scanning Probe Microscopy
SRB	Sulforhodamine B
TEM	Transmission Electron Microscopy
TNF-α	Tumor Necrosis Factor-alpha
TRIS	Tris(hydroxymethyl)aminomethane
UV-Visible	Ultraviolet–visible
VB	Vogel–Bonner
WHO	World Health Organization
%	Percent
°C	Degrees Celsius
cm	Centimeter
g	Gram
hr	Hour
kV	Kilovolt
**λ**	Wavelength
L	Liter
mA	Milliampere
mL	Milliliter
mM	Millimolar
Mm	Millimeter
μg	Microgram
μL	Microliter
μM	Micromolar (concentration)
Nm	Nanometer
pH	Potential of Hydrogen (acidity or alkalinity)
rpm	Revolutions Per Minute
V	Voltage
R^2^	Coefficient of determination, a measure of linear regression
