# Supplementary material for: Green-Synthesized Mesoporous Silica-Enhanced Herbal Nanofibers with Antimicrobial, Anti-Inflammatory, and Biocompatible Wound Healing Potential
Source: Molecules. 2026 Sep 14;31(18):3257. doi: 10.3390/molecules31183257 (PMC13609315; doi:10.3390/molecules31183257)
Supplement: Supplementary file 1 [file molecules-31-03257-s001.zip › molecules-4480330-supplementary.pdf]

Green-Synthesized Mesoporous Silica-Enhanced Herbal Nanofibers with Antimicrobial, Anti-Inflammatory, and Biocompatible Wound Healing Potential

Hebah Ayash [1,*], Gülşah Esen [2], Ece Sabuncu [2], İnci Deniz [3], Pınar Akkuş Süt [4], Gökçen Yaşayan [5], Hande Sipahi [2,6], Ahmet Aydın [2], Soodabeh Davaran [7,8] and Muhammed Hamitoğlu [2,*]

[1] Pharmacological and Diagnostic Research Center, Faculty of Pharmacy, Al-Ahliyya Amman University, Amman 19328, Jordan
[2] Department of Pharmaceutical Toxicology, Faculty of Pharmacy, Yeditepe University, 34755 Istanbul, Türkiye; gulsah.esen@yeditepe.edu.tr (G.E.); ece.sabuncu@std.yeditepe.edu.tr (E.S.); hande.sipahi@sbu.edu.tr (H.S.); ahmet.aydin@yeditepe.edu.tr (A.A.)
[3] Yağmur Sönmez Microbiology R&D Laboratory, Department of Pharmaceutical Microbiology, Faculty of Pharmacy, Yeditepe University, 34755 Istanbul, Türkiye; inci.deniz@yeditepe.edu.tr
[4] TEM & SEM & AFM Imaging Laboratory, Department of Genetics and Bioengineering, Yeditepe University, 34755 Istanbul, Türkiye; pinar.akkus@yeditepe.edu.tr
[5] Department of Pharmaceutical Technology, Faculty of Pharmacy, Yeditepe University, 34755 Istanbul, Türkiye; gokcen.yasayan@yeditepe.edu.tr
[6] Department of Pharmaceutical Toxicology, Hamidiye Faculty of Pharmacy, University of Health Sciences, 34668 Istanbul, Türkiye
[7] Drug Applied Research Center, Tabriz University of Medical Sciences, Tabriz 51656-65811, Iran; s.davaran2018@gmail.com
[8] Engineered Biomaterial Research Center, Khazar University, AZ1096 Baku, Azerbaijan
* Correspondence: h.ayash@ammanu.edu.jo (H.A.); mohammad.saz@yeditepe.edu.tr (M.H.)

Supplementary data

Table S1. The different polymer blends' preparation ratios, methods, active agents added and electrospinning parameters.

Mixture (M)	Composition	Active agents' addition	Electrospinning parameters
M1	5% total solids CS: PVA (20:80)	_	• Distance: 10cm<br>• Flow rate: 1.5mL/hr<br>• Voltage: 32kV
M2	3% total solids CS: PVA (50:50)	_	• Distance: 10cm<br>• Flow rate: 2mL/hr<br>• Voltage: 24kV
M3	1% CS and 4% PVA	_	• Distance: 12cm<br>• Flow rate: 1mL/hr<br>• Voltage: 24kV
M4	1% CS and 4% PVA	_	• Distance: 12cm<br>• Flow rate: 1mL/hr<br>• Voltage: 24kV
M5	3% CS: 5%PVA (50:50)	_	• Distance: 12cm<br>• Flow rate: 1mL/hr<br>• Voltage: 24kV
M6	3% CS: 8%PVA (50:50)	• M6a: 2mL HP ethanolic extract was added to 30 mL of the mixture	• Distance: 12cm<br>• Flow rate: 0.5mL/hr<br>• Voltage: 26kV
M7	3% CS: 8%PVA (50:50)	• M7a: 5% HP ethanolic extract of total polymer content was added to the mixture.	• Distance: 12cm<br>• Flow rate: 0.5mL/hr<br>• Voltage: 26kV
M8	1% CS: 6%PVA (50:50)	• M8a: 1% of MSNs of the total polymer content was added to the mixture.<br>• M8b: 0.5% of MSNs of the total polymer content was added to the mixture.	• Distance: 15cm<br>• Flow rate: 1mL/hr<br>• Voltage: 21.5kV
M9	2% CS: 7%PVA (50:50)	• M9a: 2mL Distilled water filtered by 0.45 µm injection filter then was added to 15mL of the mixture.<br>• M9b: 1% of MSNs of the total polymer content was dissolved in 2mL Distilled water and filtered by 0.45 µm injection filter then was added to 15mL of the mixture.<br>• M9c: 5% of HP extract powder of the total polymer content was dissolved in 2mL of Distilled water and filtered by a 0.45 µm injection filter, then added to 15ml of the mixture.<br>• M9d: 1% of MSNs and 5% HP extract powder of the total polymer content was dissolved in 2mL Distilled water and filtered by 0.45 µm injection filter then was added to 15mL of the mixture.<br>• M9e: 5% of HP oil extract of the total polymer content was dissolved in 2mL of Acetone as a co-solvent for the oil before being mixed with the polymer solution.	• Distance: 15cm<br>• Flow rate: 1mL/hr<br>• Voltage: 21.5kV
M10	2% CS: 8%PVA (30:70)	• M10a: 2mL Methanol filtered by 0.45 µm injection filter then was added to 20mL of the mixture.<br>• M10b: 1% of MSNs of the total polymer content was dissolved in 2mL Methanol and filtered by a 0.45 µm injection filter, then added to 20mL of the mixture.<br>• M10c: 5% of HP extract powder of the total polymer content was dissolved in 2mL of Methanol and filtered by a 0.45 µm injection filter, then added to 20mL of the mixture.<br>• M10d: 1% of MSNs and 5% of HP extract powder of the total polymer content was dissolved in 2mL Methanol and filtered by 0.45 µm injection filter then was added to 20mL of the mixture.	• Distance: 15cm<br>• Flow rate: 1mL/hr<br>• Voltage: 21.5kV
M11	2% CS: 8%PVA (30:70)	• M11a: 25% of HP extract powder of the total polymer content was dissolved in 5mL of Methanol and centrifuged, then the supernatant added to 10 mL of the mixture.<br>• M11b: 1% of MSNs and 25% of HP extract powder of the total polymer content was dissolved in dissolved in 5mL of Methanol and centrifuged, then the supernatant added to 10mL of the mixture.	• Distance: 15cm<br>• Flow rate: 1mL/hr<br>• Voltage: 21.5kV
M12	3% CS: 12%PVA (30:70)	• M12a: HP extract powder containing 5% hypericin of the total polymer content was dissolved in 2mL of Methanol and	• Distance: 15cm<br>• Flow rate: 1mL/hr

		centrifuged, then the supernatant was added to 10mL of the mixture.<br>• M12b: HP extract powder contains 5% hypericin and 1% of MSNs of the total polymer content was dissolved in 2mL of Methanol and centrifuged, then the supernatant was added to 10mL of the mixture.<br>• M12c: 15% of HP oil extract of the total polymer content was added to 10mL of the mixture drop by drop while stirring.	• Voltage: 21.5kV
M13	12%PVA: 3%CS: 10%G (70:15:15)	• M13a: 100mg of HP extract powder in Methanol then centrifuged, the supernatant was added to 10mL of the mixture.<br>• M13b: similar to M13a with the addition of 1% MSNs of the total polymer content.<br>• M13c: 5% herbal loaded silica 10mL of the mixture.	• Distance: 15cm<br>• Flow rate: 1mL/hr<br>• Voltage: 21.5kV
M14	12%PVA: 3%CS: 10%G (60:15:25)	–	• Distance: 15cm<br>• Flow rate: 1mL/hr<br>• Voltage: 21.5kV
M15	12%PVA: 3%CS: 10%G (70:15:15)	• M15a: 100mg of HP extract powder was dissolved in 10 mL Methanol, sonicated for 30min and left to settle for 24hr. After centrifuge the supernatant was transferred into a beaker where the methanol was evaporated at 50 °C. 10mL of the mixture is added after evaporation and mixed with a magnetic stirrer.<br>• M15b: similar to M1a with the addition of 1% MSNs of the total polymer content.	• Distance: 15cm<br>• Flow rate: 1mL/hr<br>• Voltage: 21.5kV
M16	12%PVA: 3%CS: 10%G (70:15:15)	• 1% of total solid AG was dissolved and added to polymers solution.<br>• M16a: 1% of MSNs of the total polymer content was added to the mixture.<br>• M16b: 100mg of HP extract powder was dissolved in 10 mLMethanol, sonicated for 30min and left to settle for 24hr. After centrifuge the supernatant was transferred into a beaker where the methanol was evaporated at 50 °C. 10mL of the mixture is added after evaporation and mixed with a magnetic stirrer.<br>• M16c: similar to M16b with the addition of 1% MSNs of the total polymer content.	• Distance: 15cm<br>• Flow rate: 0.7mL/hr<br>• Voltage: 21.5kV

Table S2. Mutagenicity of the sample in *Salmonella typhimurium* TA98 and TA100 strains.

Without S9		Number of revertant/plate	
		TA98	TA100
Negative control (dH2O2)		25.0 ± 4.2	193.0 ± 18.4
Positive control		822.5 ± 85.6*	1065.5 ± 54.4*
Dose (µg/plate)	100%	22.5 ± 4.8	184.0 ± 11.4
	50%	27.0 ± 1.0	178.0 ± 19.0
	25%	23.0 ± 6.5	190.5 ± 14.5
With S9			
Negative control (dH2O2)		35.2 ± 7.5	173.7 ± 19.4
Positive control		721.7 ± 23.4*	696.5 ± 42.4*
Dose (µg/plate)	100%	33.4 ± 1.5	165.3 ± 12.9
	50%	37.3 ± 4.0	177.0 ± 14.0
	25%	36.0 ± 3.6	184.3 ± 10.5

Dunnett's multiple comparison test was carried out for statistical analysis. NPD (20 µg/plate) and SA (1 µg/plate) were used as positive mutagen without S9 activation for TA98 and TA100, respectively. 2-aminofluorene (10 µg/plate) was used as positive mutagen with S9 experiment for both strains. *$p < 0.05$ versus negative control.

a

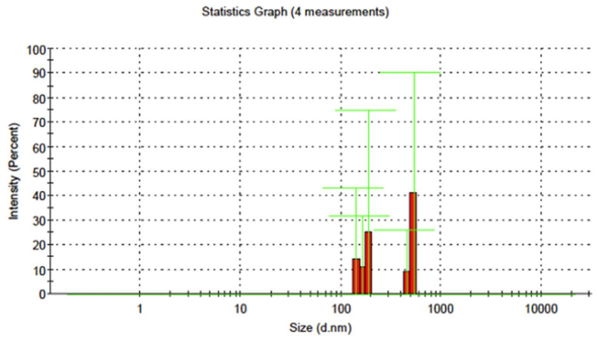

b

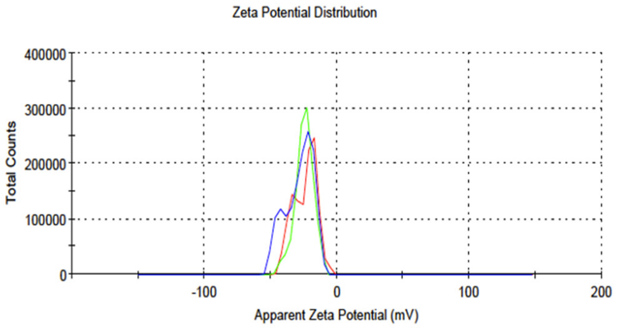

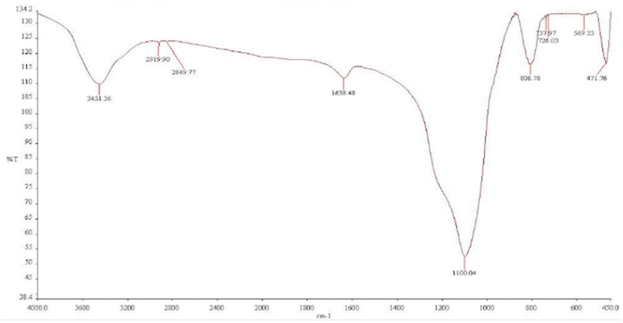

c

Figure S1. (a) Particle size intensity of MSNs (b) Zeta measurements results, and (c) FTIR spectrum of MSNs

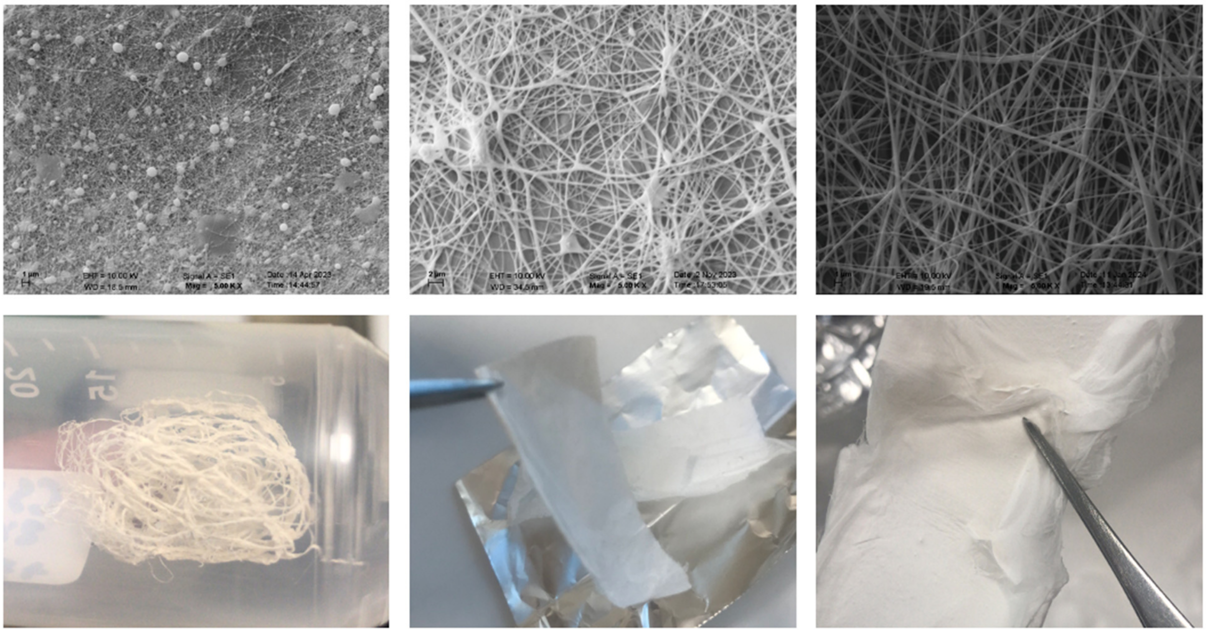

M12 M15 M16

Figure S2. Representative SEM images at 5K x of different formulations and the corresponding physical morphology

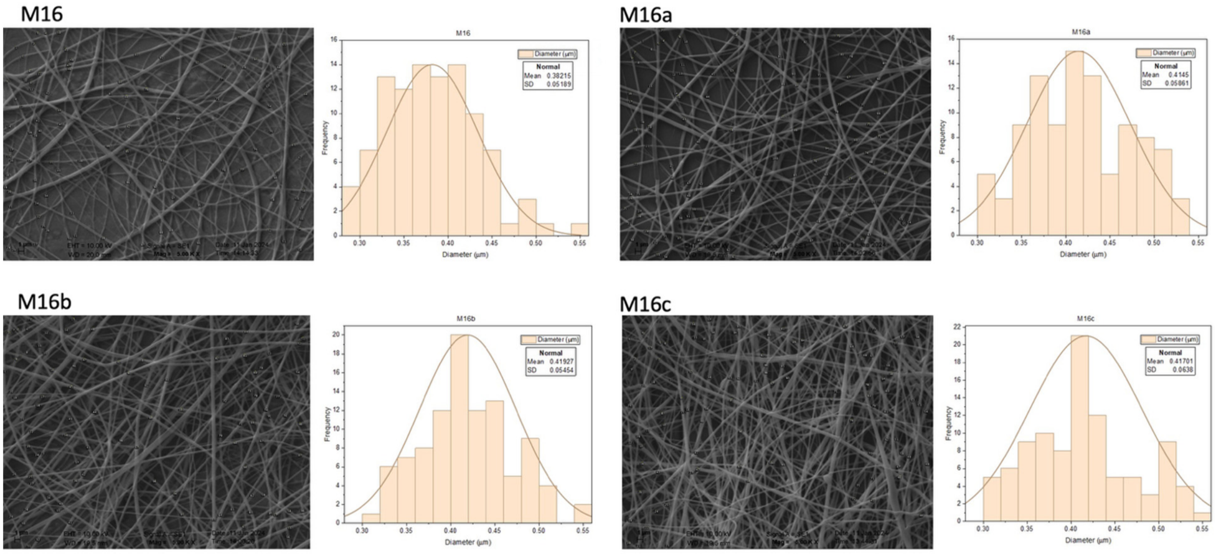

Figure S3. Fiber diameter distributions were determined from the SEM images using ImageJ for all M16 formulations

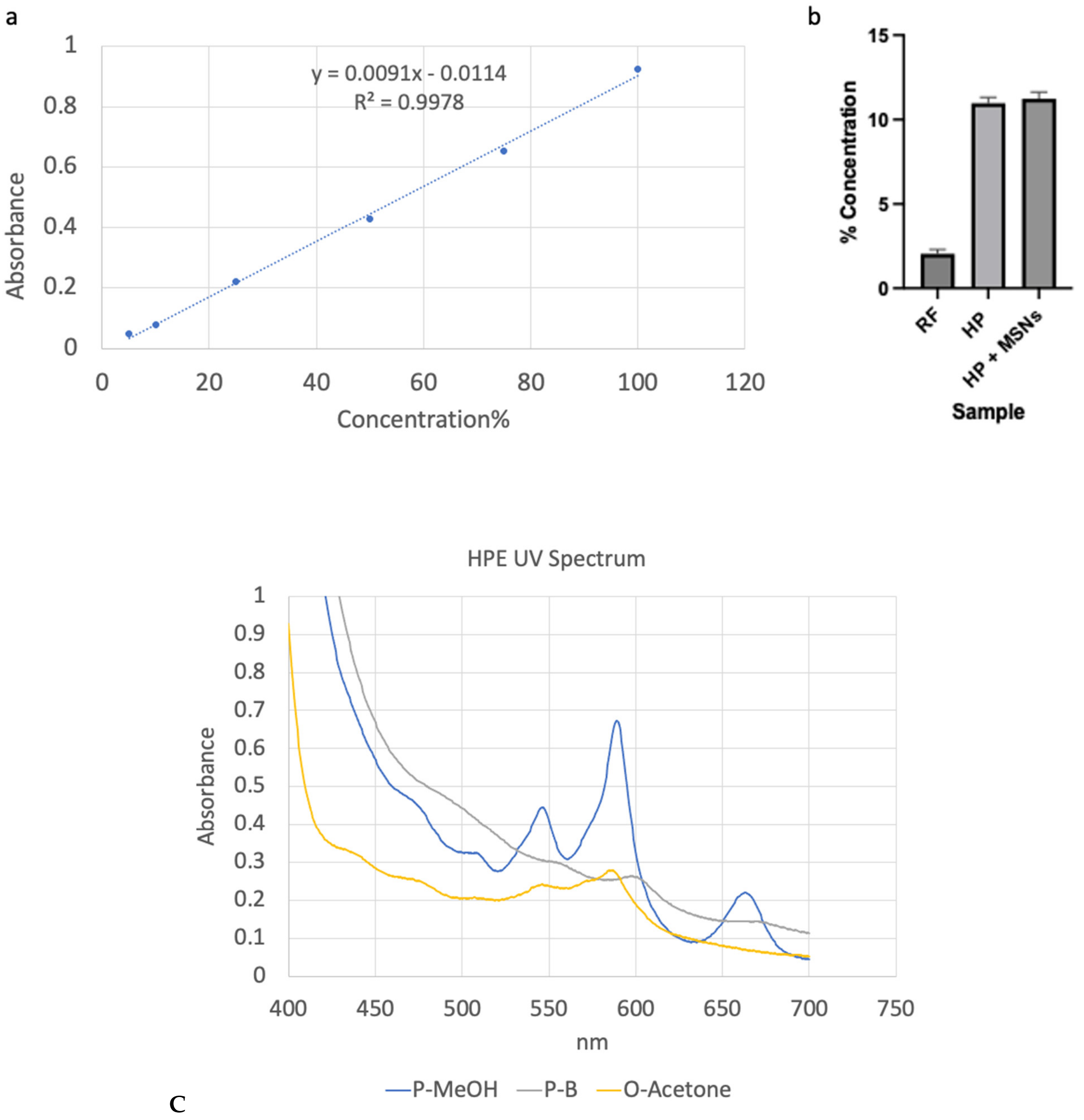

Figure S4. Hypericin absorbance spectrum and encapsulation efficiency studies. (a) Hypericin calibration curve after sample preparation (b) Actual calculated hypericin content. *p <0.0001 HP and HP + MNNs compare to the RF: raw fiber. (c) Hypericin absorbance spectrum (P: extract powders in methanol (P-MeOH) and buffer (P-B) and extract oil in acetone (O-Acetone))

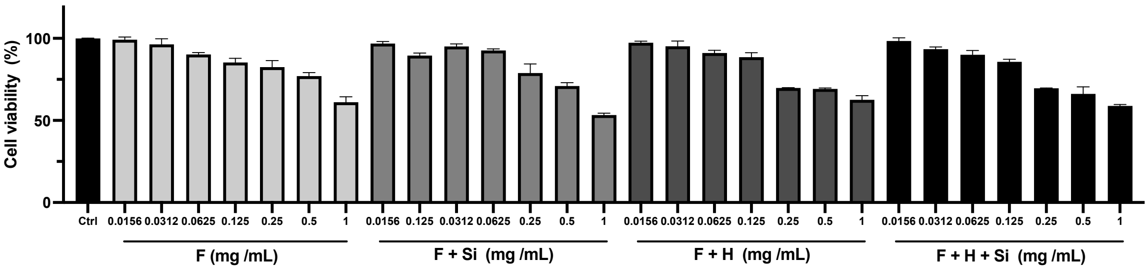

Figure S5. Cell viability results on L929 cell lines

* F: Raw nanofibers, F + Si: MSN-loaded nanofibers, F + H: Herbal-loaded nanofibers; F + H + Si: MSN and Herbal-loaded nanofibers. Values in bold indicate a significant decrease when compared to medium control.
